# Parallel Multi-Omics in High-Risk Subjects for the Identification of Integrated Biomarker Signatures of Type 1 Diabetes

**DOI:** 10.3390/biom11030383

**Published:** 2021-03-04

**Authors:** Oscar Alcazar, Luis F. Hernandez, Ernesto S. Nakayasu, Carrie D. Nicora, Charles Ansong, Michael J. Muehlbauer, James R. Bain, Ciara J. Myer, Sanjoy K. Bhattacharya, Peter Buchwald, Midhat H. Abdulreda

**Affiliations:** 1 Diabetes Research Institute, University of Miami Miller School of Medicine, Miami, FL 33136, USA; o.alcazar@miami.edu (O.A.); lfh34@miami.edu (L.F.H.); 2 Biological Sciences Division, Pacific Northwest National Laboratory, Richland, WA 99354, USA; ernesto.nakayasu@pnnl.gov (E.S.N.); carrie.nicora@pnnl.gov (C.D.N.); Charles.Ansong@pnnl.gov (C.A.); 3 Duke Molecular Physiology Institute, Duke University Medical Center, Durham, NC 27701, USA; michael.muehlbauer@duke.edu (M.J.M.); james.bain@duke.edu (J.R.B.); 4 Department of Ophthalmology, University of Miami Miller School of Medicine, Miami, FL 33136, USA; cxm1496@miami.edu (C.J.M.); sbhattacharya@miami.edu (S.K.B.); 5 Miami Integrative Metabolomics Research Center, University of Miami Miller School of Medicine, Miami, FL 33136, USA; 6 Department of Molecular and Cellular Pharmacology, University of Miami Miller School of Medicine, Miami, FL 33136, USA; 7 Department of Surgery, University of Miami Miller School of Medicine, Miami, FL 33136, USA; 8 Department of Microbiology and Immunology, University of Miami Miller School of Medicine, Miami, FL 33136, USA

**Keywords:** omics, multi-omics, metabolomics, proteomics, lipidomics, transcriptomics, biomarkers, biomarker signature, integrated analysis, network prediction, signaling pathways, type 1 diabetes, early prediction, diagnosis, prognosis

## Abstract

Background: Biomarkers are crucial for detecting early type-1 diabetes (T1D) and preventing significant β-cell loss before the onset of clinical symptoms. Here, we present proof-of-concept studies to demonstrate the potential for identifying integrated biomarker signature(s) of T1D using parallel multi-omics. Methods: Blood from human subjects at high risk for T1D (and healthy controls; *n* = 4 + 4) was subjected to parallel unlabeled proteomics, metabolomics, lipidomics, and transcriptomics. The integrated dataset was analyzed using Ingenuity Pathway Analysis (IPA) software for disturbances in the at-risk subjects compared to controls. Results: The final quadra-omics dataset contained 2292 proteins, 328 miRNAs, 75 metabolites, and 41 lipids that were detected in all samples without exception. Disease/function enrichment analyses consistently indicated increased activation, proliferation, and migration of CD4 T-lymphocytes and macrophages. Integrated molecular network predictions highlighted central involvement and activation of NF-κB, TGF-β, VEGF, arachidonic acid, and arginase, and inhibition of miRNA Let-7a-5p. IPA-predicted candidate biomarkers were used to construct a putative integrated signature containing several miRNAs and metabolite/lipid features in the at-risk subjects. Conclusions: Preliminary parallel quadra-omics provided a comprehensive picture of disturbances in high-risk T1D subjects and highlighted the potential for identifying associated integrated biomarker signatures. With further development and validation in larger cohorts, parallel multi-omics could ultimately facilitate the classification of T1D progressors from non-progressors.

## 1. Introduction

Insulin-dependent diabetes (i.e., type 1 diabetes, T1D) has afflicted humans since ancient history. For centuries, it remained a mysterious disease—causing emaciation and eventual death. It was not until the 1800s when for the first time a dietary intervention was used by Apollinaire Bouchardat (1806–1886) as a means to manage T1D [1]. Then, in the early 1900s, insulin, the first life-saving miracle drug, was discovered, and its use began to control the disease [2]. Since then, remarkable progress has been made in the understanding of the pathophysiology of T1D, and yet it inexplicably remains without a preventive or curative treatment to this day. This is likely due to the heterogeneity and complex pathogenic mechanisms of the disease, which we do not fully understand and are unable to effectively manage within the frame of the current technology. 

Perhaps the main conceptual advances in understanding T1D were (a) the adoption of the immune paradigm and (b) the extension of the metabolic disease concept to include other metabolic disturbances besides that of glucose. On one hand, the immune paradigm established the inflammatory reaction caused by immune cells and antibodies as the direct cause of the destruction of the insulin-producing β cells in the endocrine pancreas through a process referred to as autoimmunity [3]. However, the immune paradigm also predicates that the immune system can protect the β cells by activating checkpoints that prevent the immune attack, similar to what happens with cancer cells that evade immune recognition and clearance [4]. This phenomenon is referred to as the anti-inflammatory reaction or immune regulation. On the other hand, the concept of metabolic disease, which initially viewed the lack of proper glucose utilization as the only metabolic disturbance [5], has expanded to include wide metabolic shifts occurring during both autoimmune and anti-inflammatory reactions, where pro- and anti-inflammatory metabolites are actively formed, respectively [6]. Subsequently, it has become widely recognized that the treatment of T1D should aim to minimize the pro-inflammatory reaction and promote immune regulation by interventions against the immune system as well as the metabolic disturbances. Therefore, there has been great interest in ‘immunometabolism’ to modulate the immune system metabolically toward immune regulation in hopes of resolving the therapeutic conundrum of autoimmune T1D [7,8]. This has, in turn, spurred numerous metabolomics studies to characterize the metabolic profile (metabolome) of T1D patients and at-risk subjects, and to identify metabolic biomarkers for diagnostic and prognostic purposes. Other omics studies have also been conducted to understand the diabetes-associated changes in the lipidome through lipidomics, the proteome (proteomics), and the transcriptome (transcriptomics), and to identify possible corresponding biomarkers. Several lipids, proteins, and genetic features (e.g., SNPs and RNAs) have been suggested as biomarkers of risk for developing T1D. However, their broad utility in predicting the disease occurrence early or in reliably discriminating between at-risk subjects who will or will not progress to symptomatic T1D and clinical diagnosis (i.e., progressors versus non-progressors) remains limited.

In addition to the ongoing omics efforts to understand the mechanisms that lead to increased susceptibility to T1D, clinical studies—such as the BABYDIAB, The Environmental Determinants of Diabetes in the Young (TEDDY), the Diabetes Prediction and Prevention (DIPP), the Diabetes Autoimmunity Study in the Young (DAISY), and the Prospective Assessment of Newborn for Diabetes Autoimmunity (PANDA) trials [9,10]—have explored other clinical parameters to assess the risk for developing T1D in children. It is estimated that the disease risk in identical tweens is 50% when one of them has T1D; the incidence of T1D among monozygotic twins can go as high as 65% by 60 years of age [11]. The presence of T1D in first-degree relatives is also considered a risk factor in 13% of cases. Thus, in diabetic children without a family history, both genetic and environmental factors likely contribute to the remaining cases of non-genetic etiology [12,13,14,15]. In addition, risk assessment is also done based on the presence of plasma autoreactive antibodies (autoantibodies) against β-cell antigens such as, pro-insulin and insulin, glutamic acid decarboxylase (GAD), tyrosine phosphatase present in insulinomas (anti-IA2), and Zinc transporter 8 (ZnT8). Curiously, while 93% of patients diagnosed with T1D show elevated levels of one or more of these antibodies [16], only~10% of subjects with a single autoantibody progress to clinical diagnosis, and some with multiple autoantibodies never do [17]. This further highlights the need for reliable biomarkers that can discriminate between progressors and non-progressors. 

As noted earlier, transcriptomics has been used to identify differentially expressed genes as biomarker candidates of T1D. More than 40 genes have been identified as biomarker candidates of elevated risk for T1D and indicators in at-risk subjects of the likelihood of progression to clinical diagnosis [18]. Close to 60 chromosomal regions have also been implicated in having increased risk [19,20,21], and several VNTRs (variable number of tandem repeats) have been reported in the insulin gene of diagnosed patients [22,23]. The most studied genes are those of the Human Leukocyte Antigen class II (HLA class II). Specifically, the HLA DR3-DQ2 and DR4-DQ8 haplotypes have been found in 50% of T1D patients diagnosed in their childhood [24,25]. A longitudinal study showed that patients with these haplotypes who progressed to clinical diagnosis exhibited differential expression in their plasma of seven other gene products (ADCY9, PTCH1, MEX3B, IL15RA, ZNF714, TENM1, and PLEKHA5) that allowed the correct prediction of seroconversion in 85% of the cases [26]. A recent study in T1D patients also found differential expression of several additional genes involved in inflammation (*IL-1b*, *IL-1R1*, *NFkB1*), autophagia (*GABARAPL2*, *FRAP1*, *CTSL*, *CTSW*), apoptosis (*BCL2L13*), DNA repair (*APEX1*, *ERCC3*, *ERCC5*), and antioxidant activity (*PRDX1*, *SOD1*) [27]. Another study in peripheral blood mononuclear cells found 450 overexpressed and 245 downregulated genes in T1D patients [28]. Furthermore, short and long noncoding RNA (ncRNA) fragments have also been implicated in T1D [29]. MicroRNAs (miRNA; 20–25 nucleotides) have been prolifically studied in T1D patients because of their involvement in the regulation of genes involved in the disease [30,31]. Other short ncRNAs, namely, small interfering RNAs (siRNA) and Piwi-interacting (P element-interacting-wimpy testis-interacting) RNAs (piRNA) have also been implicated in T1D [32,33,34,35], and some of them appear to have a remarkable influence on its pathogenesis. For example, fragments of the miRNA200 family promoted T1D, and their suppression prevented it in animal models [36]. In subjects with high risk of T1D, the decrease in miRNA146 in peripheral blood mononuclear cells was implicated in progression toward clinical diagnosis [37]. miRNA25 was shown to be inversely correlated with the β-cell function [38], and dysregulation of miR-125b-5p, miR-365a-3p, miR-7-1-3p, miR-193a-5p, miR-200c-3p, miR-5190, miR-770-5p miR-6799-3p, miR-6793-5p, and miR-1228-3p was associated with changes in glycated hemoglobin A1C (HbA1c) in diabetic patients [39]. The plasma levels of miRNA21 and miRNA210 were also significantly higher in the plasma and urine of T1D patients [40]. Interestingly, 27 other identified miRNAs have also been associated with the function of T regulatory (Treg) lymphocytes that protect the β cells amid the autoimmune reaction [35].

Consistent with the changes described above in subjects at-risk for or diagnosed with T1D, many metabolic pathways have also been shown to be affected as well. Notably, the metabolic consequences of the lack of insulin in diabetic patients are not restricted to hindered glucose utilization in peripheral tissues; insulin insufficiency also results in abnormal levels of plasma amino acids, among other metabolites. For instance, leucine, isoleucine, phenylalanine, tyrosine, and valine levels are significantly increased, and those of glycine, glutamate, and threonine are decreased [41]. Metabolomics analyses in urine samples of T1D patients also showed high levels of glucocorticoids and mineralocorticoids, phenylalanine, tryptophan catabolites (namely kynurenine and its byproducts), glycerophospholipids, and fatty acids [42]. Low plasma levels of methionine are also correlated with elevated risk of T1D in children [43]. Moreover, proteomics studies have also shown significant changes in plasma protein levels in patients with T1D. For example, alpha-1-microglobulin, alpha-2-macroglobulin, beta-2-glycoprotein I, Ig alpha-2 chain C region, apolipoprotein A-II, and prothrombin are elevated [44,45]. Combined proteomics and ATAC-seq transcriptomics analyses in β cells treated with IFNα also showed activated genes and metabolic pathways consistent with T1D [46,47]. Notably, the lipidome is among the most investigated biochemical compartment in children with high risk of T1D [48], and lipidomics studies have shown significantly elevated plasma triglycerides and phospholipids containing polyunsaturated fatty acids in at-risk children [43,49]. Retrospective analysis of the lipidome in serum obtained from the umbilical cord of babies who were then followed up for several years showed that one lysophosphatidylcholine (LPC), two phosphatidylcholine (PC), two triglycerides, and two unidentified lipid species were good predictors of T1D risk in these infants [50,51,52]. Moreover, other studies revealed a decrease in plasma sphingomyelins (SM) in high-risk T1D children [53,54].

Taken together, it is evident that substantive omics efforts have been expended to understand the pathogenic mechanisms of T1D and to identify potential metabolic, protein, lipid, and genetic biomarkers of the disease. However, the fact remains that there is currently no coherent picture of what constitutes a reliable biomarker or combination of biomarkers that allow clinically implementable (a) prediction of T1D in at-risk subjects before the manifestation of symptoms and (b) discrimination between progressors and non-progressors. It is also becoming clear that the combination of multiple omics (multi-omics) and the application of data mining techniques in the analysis of the associated multiple datasets is more promising than single omics in providing a more comprehensive picture of the pathophysiology of T1D and identifying associated biomarker signature(s). It should be noted, however, that most studies designated as “multi-omics” so far have been restricted to only two omics-type analyses typically conducted in different sets of samples [43,46,52]. Recently, there have been a couple of reports on the integration of multiple datasets obtained from multiple platforms [55,56]. A composite immune signature for T1D progression has been suggested [57]. However, to the best of our knowledge, there are no integrated multi-omics studies in the context of T1D. In this report, we present for the first-time proof-of-concept quadra-omics analyses (metabolomics, proteomics, lipidomics, and transcriptomics) in plasma samples of subjects with high risk for developing T1D compared to healthy controls. Notably, these multi-omics studies, performed in aliquots of the same samples, demonstrated the feasibility of conducting such parallel quadra-omics studies and integrating the analyses in the context of T1D. We also report on technical aspects of the integrative analysis and discuss important considerations when working with individual or combined datasets obtained through parallel multi-omics.

## 2. Materials and Methods

### 2.1. Sample Collection

Blood samples (~20 mL/subject in EDTA) were collected at the Diabetes Research Institute of the University of Miami from consented male/female subjects (*n* = 4) considered at high risk for T1D during routine visits as part of the ongoing TrialNet’s Natural History Study of the Development of Type 1 Diabetes (Pathway to Prevention Study) TN-01 study. Subjects in the TN-01 study Living Biobank are tested semi-annually for the appearance of new or additional autoantibodies and evaluated metabolically by oral glucose tolerance test (OGTT) to assess their progression toward clinical diagnosis of T1D. Samples from healthy subjects (*n* = 4) were collected as part of another study approved by the IRB of the University of Miami (study number 11995-115). Plasma was obtained immediately after blood collection and stored at −80 °C until analysis. All samples were analyzed within 2 months of collection in a blinded fashion. These trials are conducted in accordance with the principles of the Declaration of Helsinki and consistent with the Good Clinical Practice guidelines of the International Conference on Harmonization. The protocol for the ancillary study, under which the current multi-omics analyses were performed, was approved by TrialNet (study ID number 195) and its IRB. The four high-risk subjects in the present report were staged for their risk level according to the TrialNet staging/scoring system, which considers family history, genetic susceptibility according to haplotype (e.g., HLA-DQ/DR), the number of autoantibodies, and OGTT results as follows: low-risk (1 autoantibody and normal OGTT); moderate-risk (2–3 autoantibodies and normal OGTT); high-risk (4–5 autoantibodies and normal OGTT); and very high-risk (4–5 autoantibodies and abnormal OGTT). During sample collection, one of the four high-risk subjects exhibited signs of abnormal OGTT and was confirmed to have converted to a new-onset patient during a second OGTT and another sample collection two weeks later. Both samples were independently analyzed by multi-omics, and the averaged values for each identified feature in both samples were included in the integrative analyses as part of the high-risk group to avoid further reduction in the subject number. See Supplementary Table S1 for demographic, serology, OGTT, and other information/parameters. Samples from all subjects were divided into four equal aliquots that were individually subjected to proteomics, metabolomics, lipidomics, and transcriptomics (miRNAs) analyses (i.e., parallel quadra-omics) that were performed in collaboration with the Miami Integrative Metabolomics Research Center (lipidomics) [58], Pacific Northwest National Laboratories (proteomics) [59], Ocean Ridge Biosciences (transcriptomics) [60], and the Stedman Metabolomics Laboratory at Duke University Medical Center (metabolomics) [61]. 

### 2.2. Proteomics Analysis 

Samples were diluted 8-fold with Agilent buffer A and filtered with a 0.22 µm centrifugal filter. Diluted samples were loaded onto Multiple Affinity Removal System (MARS) column (Hu-14 4.6 × 100 mm, Agilent Technologies, Santa Clara, CA, USA) coupled with a 1200 series HPLC (Agilent, Santa Clara, CA, USA) to deplete the 14 most abundant plasma proteins (albumin, IgG, α1-antitrypsin, IgA, IgM, transferrin, haptoglobin, α1-acid glycoprotein, α2-macroglobulin, apolipoprotein A-I, apolipoprotein A-II, complement C3, transthyretin and fibrinogen). The column was re-equilibrated for 13 min at 1 mL/min with Agilent buffer A. Samples were analyzed with a 27 min gradient (18 min sample load at 0.125 mL/min and 2 min wash at 1 mL/min both with Agilent buffer A, followed by bound protein elution for 7 min at 1 mL/min Agilent buffer B). Unbound fraction, containing low- and mid-abundant proteins, had its buffer exchanged to 50 mM Tris-HCl, pH 8.0 using 3-kDa molecular mass cutoff Amicon centrifugal filters (Millipore, Burlington, MA, USA). Protein concentration was determined by BCA assay (Thermo Scientific, San Jose, CA, USA) for quality control. Urea pellets and 500 mM dithiothreitol (both from Sigma-Aldrich, St. Louis, MO, USA) were added to final concentrations of 8M and 5 mM, respectively. Disulfide bounds were reduced by incubating for 1 h at 37 °C. Reduced cysteine residues were alkylated by adding 500 mM iodoacetamide to a final concentration of 10 mM and incubating for 45 min at 25 °C in dark. Proteins were digested for 2 h at 25 °C using 1:50 (enzyme/protein ratio) of sequencing-grade endoproteinase Lys-C, followed by overnight incubation with 1:50 sequencing-grade trypsin at 25 °C. Digestion was quenched by adding pure formic acid to a final concertation of 1%. Peptides were desalted by solid phase extraction using tC18 Sep Pak cartridges (Waters, Milford, MA, USA) and dried in a vacuum centrifuge (ThermoFisher Scientific, Carlsbad, CA, USA). 

Samples were derivatized with a 10-plex tandem mass tag (TMT) kit (ThermoFisher Scientific) and fractionated into 24 fractions by high-pH reversed phase chromatography as previously described [62]. BCA assay was performed after peptide labeling to check for losses during sample preparation. LC-MS/MS analysis was done on a Waters NanoAquity UPLC system with a custom packed C18 column (70 cm × 75 μm i.d., Phenomenex Jupiter, 3 μm particle size, 300 Å pore size) coupled to a Q-Exactive mass spectrometer (Thermo Fisher Scientific). Peptides were eluted with the following gradient of water (solvent A) and acetonitrile (solvent B) both containing 0.1% formic acid as follows: 1–8% B in 2 min, 8–12% B in 18 min, 12–30% B in 55 min, 30–45% B in 22 min, 45–95% B in 3 min, held for 5 min in 95% B and 99–1% B for 10 min. Analytes were analyzed online by nanoelectrospray ionization. Full mass scans were collected from 300 to 1800 *m/z* at a resolution of 35,000 at 400 *m/z*. Tandem mass spectra were collected in data-dependent acquisition of the top 12 most intense parent ions using high-energy collision induced dissociation (HCD) fragmentation (2.0 *m/z* isolation width; 30% normalized collision energy; 17,500 resolution at 400 *m/z*), before being dynamically excluded for 30 s. A quality control sample, which consists of a protein digest of the bacterium *Shewanella oneidensis*, was also run before and after each batch of samples for additional quality control following a routine procedure [63].

Data were extracted with Decon2LS_V2 and DTA Refinery [64,65] for mass recalibration and peak list extraction. Identification of peptides were done with MSGF+ [66] using the human SwissProt database (downloaded from Uniprot Knowledgebase on 22 February 2019). The analysis parameters were: parent ion mass tolerance of ±6 ppm, tryptic digestion in at least one of the peptide termini, 2 missed cleavages allowed, cysteine carbamidomethylation (+57.0215 Da) and N-terminal/lysine TMT labeling (+229.1629 Da) derivatization as static modifications, and the variable modifications: oxidation (+15.9949 Da) on methionine, cysteine, tyrosine and tryptophan; dioxidation (+31.9898 Da) on cysteine; and deamidation/deamination (+0.98402 Da) on asparagine, glutamine and arginine residues. Spectral-peptide matches, peptides and protein were filtered with MSGF probabilities to maintain false-discovery rate below 1% in each of the levels. The intensities of TMT reporter ions were extracted with MASIC [67] for quantitative analysis.

### 2.3. Metabolomics Analysis

Non-targeted metabolomics of plasma samples were conducted via gas chromatography/ electron-ionization mass spectrometry (GC/EI-MS) using the method from [68]. Plasmas were extracted by the addition of methanol spiked with perdeuterated myristic acid standard (used for adjusting GC column pressure for consistent retention time), dried by SpeedVac, and derivatized by methoximation and trimethylsilylation. All samples were run in a single batch with a process blank (ghost) for quality control. The ghost consisted of a blank sample that was processed similar to the experimental and included in the batch run for tracking impurities. All samples were run on a 7890B GC-5977B EI-MS (Agilent Corporation; Santa Clara, CA, USA) with MS scans set broadly from *m/z* 50 to 600 during a GC heat ramp spanning from 60 to 325 °C. The instrument was appropriately tuned prior to running the samples (including ghost) and the process blank/ghost was used to identify and discard any chromatographic features considered process contaminants. Deconvoluted spectra were annotated as metabolites using an orthogonal approach incorporating both the GC retention time (RT) and the MS fragmentation pattern. Peak annotation was based primarily on an RT-locked spectral library of metabolites, built upon the Fiehn GC/MS Metabolomics RTL Library [69]. In this discovery protocol, chromatographic features were evaluated as log-2 transformed areas under the curve (log 2 AUC) to represent feature abundance. Additional metabolite features that could not be annotated from the library were found by SpectConnect [70] matching spectral similarities between samples.

### 2.4. Lipidomics Analysis

Lipids were extracted from 50 µL of plasma by adding 4 mL of methyl tert-butyl ether (MTBE) and 1.2 mL of butylated hydroxytoluene (BHT) then incubating overnight at 4 °C. The following day, 1.25 mL of 0.15 M ammonium acetate was added, and the samples were centrifuged at 2000× *g* for 10 minutes at 4 °C (Thermo Fisher Scientific Megafuge 8R) to obtain phase separation. The upper organic phase was collected and the extracted lipids equivalent to 100 µg of protein were dried in a speed vacuum concentrator (Labconco). The samples were reconstituted in 50 µL of chloroform:methanol (1:1) and sonicated before analysis by liquid chromatography tandem mass spectrometry (LC-MS/MS). The lipids were analyzed by LC-MS/MS using an Accela 600 HPLC (Thermo Fisher Scientific) and a Q Exactive Orbitrap mass spectrometer (Thermo Scientific) as previously described [58]. The lipids were separated on an Acclaim™ 120 C18 column (Thermo Fisher Scientific) with a flow rate of 260 µL/min. The column temperature was 30 °C and the tray temperature was 20 °C. Solvent A was composed of 10 mM ammonium acetate in 60:40 methanol:water with 0.1% FA. Solvent B was composed of 10 mM ammonium acetate with 60:40 methanol: chloroform with 0.1% FA. The gradient was 35–100% solvent B over 13.0 min, 100% solvent B over 13.0–13.8 min, 100–35% solvent B over 13.8–14.5 min, 35% solvent B over 14.5–18.0 min, 0% solvent B over 18.0–20.0 min. All solvents were LC-MS/MS grade. The heated electrospray ionization (HESI) source was operated in both positive and negative polarities. The sheath gas was set to 45 and 30 for positive and negative mode, respectively. The auxiliary gas was set to 15 (positive mode) and 14 (negative mode). The spray voltage was 4.4 kV and the s-lens radio frequency (RF) level was 70. Full MS scans were acquired using a resolution of 70,000, automatic gain control (AGC) target of 1 × 10^6^ ions, and maximum injection time of 100 ms. For quality control, blanks and control samples consisting of bovine liver extract spiked with known concentrations of deuterated standards are routinely run between experimental samples. Data-dependent MS/MS scans were acquired using a resolution of 17,500, AGC target of 1 × 10^5^ ions, and maximum injection time of 50 ms. The normalized collision energies (NCE) were 15, 30, 45, 60, 75, and 90. Raw files were analyzed in Lipid Search 4.1 (a Thermo Scientific proprietary database built into their software) to identify and quantify the lipids. A product search was performed in Lipid Search with the following search parameters: product *m*/*z* tolerance 5 ppm; *m*/*z* tolerance 5 ppm; quantification: *m*/*z* tolerance 5 ppm, retention time tolerance 1 min. All classes were selected and searched. The following adducts were selected in positive mode: +H, +NH_4_, +H-H_2_O, +H-2H_2_O, +2H, +Na, +K. The following adducts were selected in negative mode: -H, +HCOO, +CH_3_COO, -2H, -Cl.

### 2.5. Transcriptomics Analysis

Human plasma samples were filtered through Costar 0.45 μm filters (Thermo Fisher Scientific, Waltham, MA, USA; Part # 07-200-388), and RNA was isolated from 300 μL of the filtrate utilizing Tri Reagent BD (Molecular Research Center, Cincinnati, OH; Part # TR118). Quality control monitoring was performed by adding a mixture of 4 synthetic microRNAs including cel-miR-257 at a mass of 2.5 fmoles per 300 μL sample (see Supplementary Table S2 for additional quality control information). After phase separation, RNA was purified using EPOCH RNA silica spin columns (EPOCH Life Science, Missouri City, TX, USA; Part # 1940-250), digested with RNase free DNase I (Lucigen, Middleton, WI, USA; Part # D9905K), and re-purified on RNeasy MinElute columns (Qiagen, Hilden, Germany; Part #: 74204) using an alternative high-ethanol binding condition to preserve low molecular weight RNA. For quality control, real-time PCR was performed on 0.87% of the total RNA sample for reverse-transcription of the spiked-in cel-mir-257 microRNA, and 2.6% of the sample for reverse transcription of each of the endogenous microRNAs (miR-150 and miR451). RNA was considered intact if expected CT values for the two endogenous microRNAs were detectable (CT < 35) and had a recovery of cel-miR-257 > 1%. For library preparation, template DNA molecules were prepared from 150 μL of plasma equivalent of the isolated low molecular weight RNA samples using the CleanTag Small RNA Library Prep Kit (TriLink Biotechnologies, San Diego, CA, USA; Part # L-3206) according to the manufacturer’s instructions. The purified libraries were electrophoresed through a 8% native acrylamide gel, and library fragments of 131–160 nt corresponding to inserts of 12-41 nt were excised from the gel and recovered by overnight agitation at 37 °C at 200 rpm in elution buffer, passage of the eluate through a 0.45 μM filter, and ethanol precipitation. The quality and size distribution of the amplified libraries were determined by chip-based capillary electrophoresis on Agilent 2100 Bioanalyzer High Sensitivity DNA assays (Agilent Technologies, Santa Clara, CA, USA; Part # 5067-4626). Libraries were quantified using the Takara Library Quantification Kit (Takara, Kyoto, Japan; Part # 638324). For sequencing, the libraries were pooled at equimolar concentrations and diluted prior to loading onto the flow cell of the Illumina NextSeq 500. The libraries were extended and bridge-amplified to create sequence clusters and sequenced with 75 nt single-end reads plus a 6 nt index read using the Illumina NextSeq 500 75 cycle high-output v2.5 sequencing kit (Illumina, San Diego, CA, USA; Part # 20024906) controlled by the NextSeq Run Manager version 2.2.1.1645. Real time image analysis and base calling were performed on the instrument using the Real-Time Analysis (RTA) software version 2.11.3. Base calls from the NextSeq 500 RTA were converted to sequencing reads in FASTQ format using Illumina’s bcl2fastq program version 2.20.0.422. Only reads passing Illumina’s quality filter were kept for analysis. All samples had a minimum of 14.65 million passed-filter 75 nt single-end reads. After removal of reads comprised substantially of adapter sequences, and trimming partial adapter sequences, the reads aligned at an average of 63 ± 15% (SD) efficiency to the human hg38 reference genome.

### 2.6. Data Analysis

Following standard curation and normalization procedures to ensure that all measured features were identified in all samples without exception, each dataset from the different independent omics analyses (namely, proteomics, metabolomics, lipidomics, and transcriptomics) was first analyzed on its own to identify significantly different features between the high-risk T1D and healthy subjects (*p* < 0.05, *t*-test with correction for multiple comparisons using the Holm–Sidak method in GraphPad Prism; GraphPad, La Jolla, CA, USA). Then, they were analyzed using the Ingenuity Pathway Analysis (IPA) software package (Qiagen Bioinformatics; Redwood City, CA, USA; https://www.qiagenbioinformatics.com/products/ingenuity-pathway-analysis; RRID:SCR_008653) [71] to identify the most significantly affected pathways, their components and associated signaling networks. Analyses in IPA were first done for each omics dataset separately and then in the combined quadra-omics dataset using an integrative approach. The analyses were conducted at various stringency levels for feature selection corresponding to five different fold-change cutoff values (1.1, 1.2, 1.3, 2.0, and 3.0) in the datasets, and then used for the prediction and visualization of associated interaction networks, canonical pathways, upstream regulators, functions and diseases enrichment, and biomarker prediction in IPA using as input the matrices of relative abundance values of the identified features annotated by their gene names where applicable.

## 3. Results

### 3.1. Parallel Untargeted Quadra-Omics

**Figure 1:**
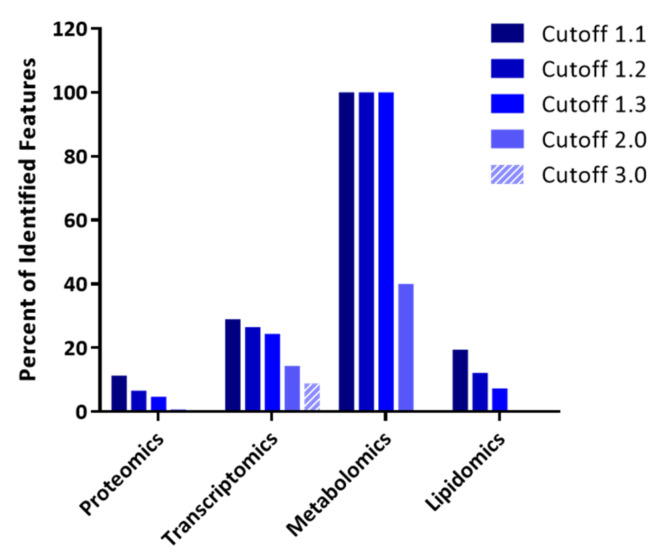
The impact of thresholding strategy on the integrative analysis of parallel multi-omics datasets. Bar graph representation of features remaining in each omics dataset after different levels of selection stringency for feature selection (fold-change cutoff value) used for input into the integrative analysis shown as percentages of the total identified features (see Table 1 for absolute numbers).

The strategy of integrating data from four different types of omics analyses performed in parallel on the same samples (herein referred to as parallel quadra-omics) is central to the present study. Human blood samples were obtained from subjects considered at high risk for developing T1D under TrialNet guidelines (see Methods) as well as from healthy individuals to serve as control. Each sample was evenly split into four aliquots for parallel, independent analyses using different untargeted omics technologies—namely, proteomics, metabolomics, lipidomics, and transcriptomics. A total of 2292 proteins, 75 small-molecule metabolites, 41 lipid species, and 328 miRNAs were identified without exception in all samples/subjects (Table 1). Data selection for analysis by increasing the threshold for feature selection based on fold-change (cutoff value) resulted in decreased number of features in the individual datasets (Table 1 and Figure 1).

**Figure 2:**
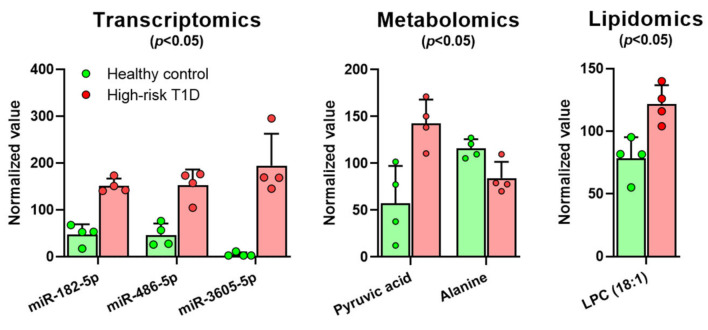
Independent analyses in the individual datasets from multi-omics reveal modest differences in the transcriptome, metabolome, and lipidome of high-risk T1D subjects compared to healthy controls with only a few significantly different features. A comparison between high-risk T1D subjects (red/pink) and healthy controls (green; *n* = 4 each), using each omics-type dataset pooled from each group, was performed by *t*-test with correction for multiple comparisons with the Holm–Sidak method. Shown are all miRNAs, lipids, and metabolites that had significant differences between the two groups (*p* < 0.05).

Comparisons within each type of the omics datasets by *t*-test (with correction for multiple comparisons using the Holm–Sidak method) indicated that a handful of miRNAs, metabolites, and lipids had significantly different abundances in the samples from the high-risk T1D subjects compared to those from the healthy controls (*p* < 0.05). Specifically, the miRNAs miR-182-5p, miR-486-5p, and miR-3605-5p; LPC(18:1); and pyruvate (pyruvic acid) were all increased, whereas alanine was decreased (Figure 2).

### 3.2. The Impact of Thresholding Strategy on the Distribution of Features Identified by Quadra-Omics

To ensure the robustness of our analyses, we evaluated results at five different levels of stringency in the feature selection corresponding to fold-change threshold values (cutoffs) that ranged from 1.1 to a maximum of 3.0. The number of features left for analysis decreased with increasing stringency in the cutoff value (see Table 1 and Figure 1), and at the highest value (3.0), this number fell well below the recommended minimum of 100 features by IPA (https://go.qiagen.com/IPA-transcriptomics-whitepaperaccessed on 16 February 2021). Additionally, information derived from two omics analyses was completely lost at this level of stringency. Hence, the results were evaluated at the cutoff values of 1.1, 1.2, 1.3, and 2.0, which all yielded the recommended minimum of total 100 features for the analysis (Table 1 and Figure 1).

Notably, while the proteomics measurements yielded the highest number of total identified features (2292), the number of proteins showing at least a 10% change (i.e., cutoff 1.1) was much less (260) and comparable to those showing such a change in the metabolomics (75) and transcriptomics (95) datasets. This might be due to the tandem mass tagging method we used to barcode the samples which can result in fold-change compression [72]. Consequently, the proteomics data were consistently the most affected by raising the stringency—i.e., the cutoff value—in the analysis compared to the other datasets, so that at a cutoff value of 2.0, only 18 proteins remained (0.8%) compared to 47 miRNAs (14.3%) and 30 metabolites (40.0%) (Table 1 and Figure 1). No lipid features remained at ≥2.0 cutoff either. A comparison of the relative impact of the applied cutoff value showed that the metabolomics dataset was the least affected and the proteomics the most (Figure 1). Nevertheless, the large number of features identified by proteomics ensured a fair number of proteins to be included in the integrative analyses even at the cutoff value of 2.0.

### 3.3. Comparative Enrichment Analyses for Diseases and Functions, Canonical Pathways, and Upstream Regulators

**Figure 3:**
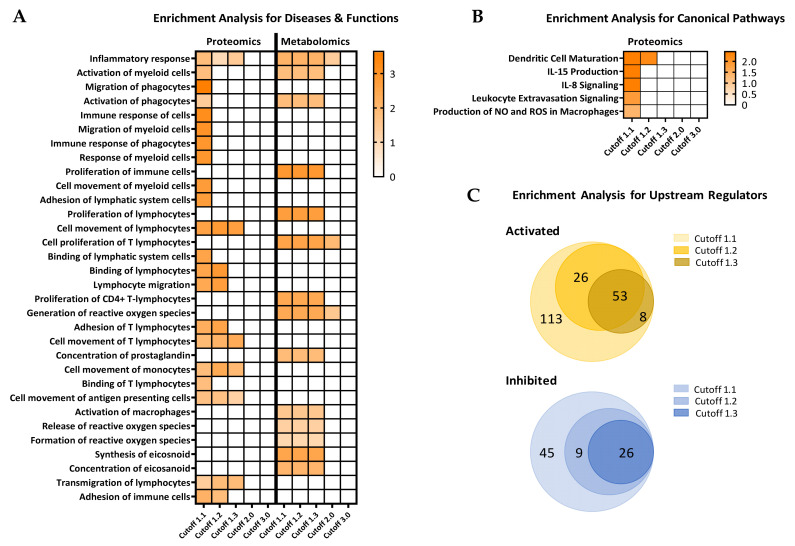
The ability to predict diseases and functions and alterations in canonical pathways and upstream regulators in high-risk T1D subjects decreases with increasing stringency of feature selection. Proteomics and metabolomics datasets with feature selection at different cutoff values were interrogated for predicted involvement in various immune inflammatory processes/pathways using the “Comparison Analysis” module of IPA software. (**A**) Diseases and functions predicted to be most affected in the high-risk T1D subjects compared to healthy controls by independent analyses in the proteomics and metabolomics datasets. (**B**) Canonical pathways analysis with increasing cutoff values for feature selection in the proteomics dataset. (**C**) Upstream regulator analysis in the proteomics, metabolomics, and lipidomics datasets, without curation for any specific physiological function at the indicated cutoff values, showing the total number of upstream regulators predicted to be activated and inhibited. Results in A and B are shown as heatmaps with the orange color indicating activation and its intensity the magnitude of the *z*-value (statistical score that accounts for the directional effect of change for functions or molecules in the experimental datasets; https://go.qiagen.com/IPA-transcriptomics-whitepaper, accessed on 16 February 2021). Results in C are shown as concentric Venn diagrams showing the number of activated (orange) or inhibited (blue) upstream regulators predicted in the analyses at the indicated cutoff values; no predictions were possible at cutoff values of 2.0 or above.

For more detailed analyses of the parallel multi-omics datasets and to exploit the advantages of performing complex enrichment and comparative analyses across pathways and signaling networks in an integrative fashion between the at-risk and healthy subjects, we performed comprehensive comparisons using the IPA software package. Initial enrichment analysis using the “Diseases & Functions” module in the proteomics and metabolomics datasets independently predicted several immune and inflammatory related functions as activated in the high-risk T1D subjects compared to healthy controls (Figure 3A). The analysis yields from each dataset were evaluated with increasing stringency for feature selection at the cutoff values of 1.1, 1.2, 1.3, 2.0, and 3.0. The largest numbers of potentially affected functions in the high-risk T1D group were obtained at the lowest cutoff value of 1.1. Biologically relevant functions and processes that were predicted to be significantly activated in these subjects (as compared to healthy controls) included: inflammatory response, proliferation of immune cells (particularly CD4+ T-lymphocytes), T-lymphocytes movement, activation of macrophages, generation of reactive oxygen species (ROS), and synthesis of eicosanoids, among others. Interestingly, several of these immune functions/processes were consistently predicted at the cutoff values of 1.1, 1.2, and 1.3, but no longer at ≥2.0 (Figure 3A; data presented as heatmap with increasing *z*-value); thus, highlighting the potential impact of the thresholding strategy on the analysis.

Further analysis using the “Enrichment Analysis for Canonical Pathways” (in IPA) with decreasing selection stringency in the proteomics dataset highlighted the potential of revealing significantly altered pathways in the high-risk T1D group based on the thresholding strategy. The activation of dendritic cell maturation pathway was predicted as the most prominently altered, followed by IL-15 production, IL-8 signaling, and the production of ROS and nitric oxide (NO) in macrophages (Figure 3B; data presented as heatmap with increasing *z*-value). Notably, at cutoff values of 1.3 and above, the IPA software ability to predict alterations in canonical pathways between the two groups was diminished. This was further highlighted in the analysis of “Upstream Regulators”, which collectively predicted 200 activated and 80 inhibited such regulators in the high-risk T1D group compared to healthy subjects (Figure 3C). Like in the enrichment analysis for diseases and functions and for canonical pathways, increasing the stringency in the analysis resulted in the progressive reduction in information yield and IPA’s ability to make predictions of upstream regulators.

### 3.4. Regulator Effects Analysis Predicting Cause-Effect Relationships in the Integrated Quadra-Omics Dataset

**Figure 4:**
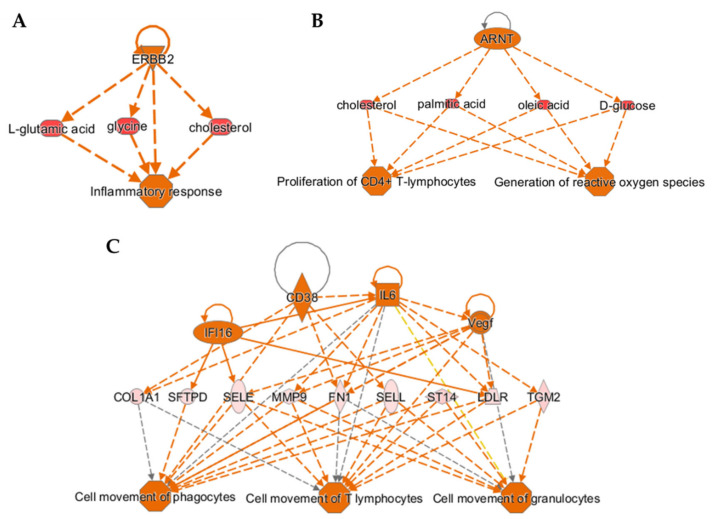
Cause-effect relationship analysis provides mechanistic insight into T1D pathogenesis based on integrated multi-omics datasets. (**A**–**C**) Three-tier diagrams (networks), predicted by the “Regulator Effects” analysis module in IPA software using the integrated quadra-omics dataset with decreasing stringency of feature selection threshold (i.e., fold-change cutoff). Each network shows the predicted upstream regulators (top row), selected molecules with significant differential expression as identified in the actual data (middle row), and the immune functions/processes predicted to be affected (bottom row). (**A**) A network that was consistently predicted at cutoff values of 1.1, 1.2, and 1.3 showing activation of the inflammatory response. (**B**) A network predicted at the cutoff values of 1.1 and 1.2 showing upregulation of CD4+ T-lymphocyte proliferation and ROS generation. (**C**) A network predicted only at the cutoff value of 1.1 showing activation of cellular movement of T-lymphocytes, granulocytes, and phagocytes. Marker key—Triangle: kinase; horizontal-oval: transcription regulator; vertical-oval: transmembrane receptor; diamond: enzyme; square: cytokine; horizontal ellipse: metabolite; circle: other; octagon: function. Color key—orange: predicted activation, blue: predicted inhibition. Connecting line color key—orange: activation; blue: inhibition; and gray: not predicted.

Taking advantage of the “Regulator Effects” analysis module in IPA, we performed network predictions in the integrated quadra-omics dataset to reveal possible cause-effect relationships and the underlying mechanisms leading to the altered proteome, metabolome, lipidome, and transcriptome of the high-risk T1D subjects as compared to healthy controls. This integrative analysis approach connects predicted putative upstream regulators with biological effects relevant to immune functions and inflammatory processes identified in the combined multi-omics dataset (Figure 4). Connecting diagrams (networks) were predicted when applying cutoff values of 1.1 through 1.3; no network predictions in this type of analysis were possible at cutoffs 2.0 and above. Consistent with the results obtained from the enrichment analysis of diseases and functions in individual datasets (Figure 3A), activation of inflammatory responses in the high-risk T1D group was apparent in the integrative regulator effects analyses performed at the cutoff values of 1.1, 1.2, and 1.3. In addition, the analyses consistently predicted upregulation of the receptor tyrosine-protein kinase *ERBB2* gene product (ERBB2), which is involved in the activation of inflammatory responses mediated by pro-inflammatory metabolites, such as glutamic acid, glycine, and cholesterol (Figure 4A) [73,74,75], and has been strongly implicated in T1D [76,77,78]. Analyses with less stringent feature selection thresholds yielded additional mechanistic insight into signaling pathways predicted to be altered in the high-risk T1D subjects. The analyses at 1.1 and 1.2 indicated the activation of proliferation in CD4+ T-lymphocytes and ROS generation in macrophages through the upregulation of aryl hydrocarbon receptor nuclear translocator (*ARNT*) gene product (also known as, HIF-1β) via the pro-inflammatory metabolites indicated in Figure 3B. Other examples of predicted networks obtained at the cutoff value of 1.1 included the activation of cellular motility in various immune cells, including T-lymphocytes, through the upregulation of pro-inflammatory signals like interleukin-6 (IL-6), cluster of differentiation 38 (CD38), interferon gamma inducible protein 16 (IFI16), and vascular endothelial growth factor (VEGF) (Figure 3C). Notably, the breadth and complexity of the predicted networks was diminished with increasing stringency of the feature selection (see Supplementary Figure S1), further highlighting the impact of the thresholding strategy in these integrative analyses.

### 3.5. Integrative Molecular Network Analysis of Parallel Multi-Omics Datasets Facilitates the Identification of Potential T1D Biomarker Signature(s) 

**Figure 5:**
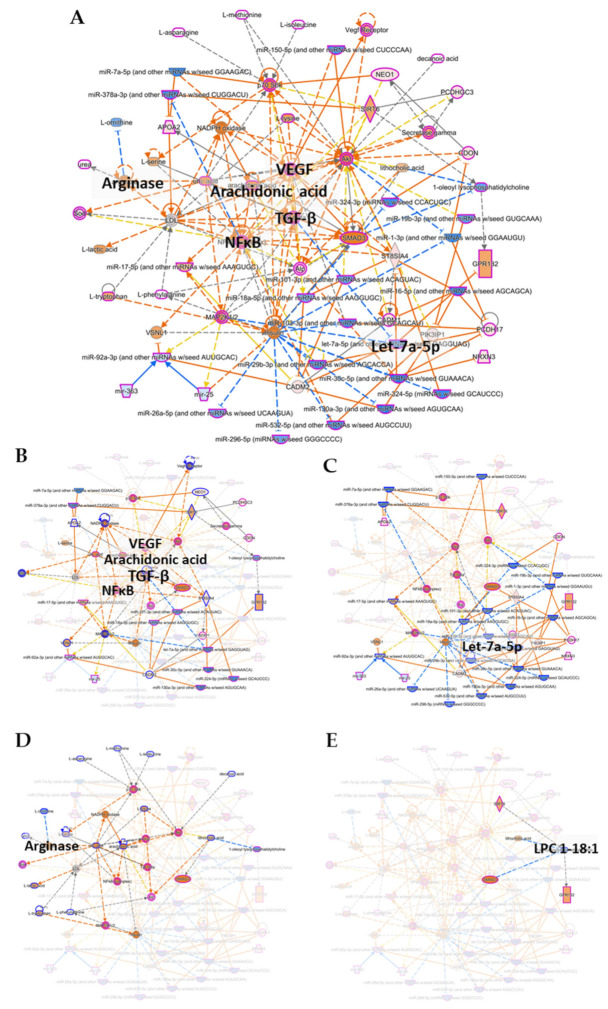
Integrated molecular network analysis in combined quadra-omics datasets highlights the potential for identifying integrated biomarker signatures of T1D. The integrated quadra-omics dataset was analyzed in IPA using the “Molecular Network Prediction” module with a focus on immune/inflammatory processes. The integrated global network shown in (**A**) was obtained at the cutoff value of 1.1 and was curated to reduce complexity and highlight nodes with known involvement in T1D, namely, NF-κB, TGF-β, VEGF, arachidonic acid, arginase, and the microRNA Let-7a-5p (Let-7) family. Individual contributions to this network by each omics-type dataset are highlighted in full color and some nodes annotated (for emphasis) with the rest of the network faded in the background (for clarity): (**B**) proteomics, (**C**) small transcriptomics, (**D**) metabolomics, and (**E**) lipidomics. Network elements are represented by symbols of various marker shapes for identification and different colors for activation or inhibition. Marker shape key—horizontal-oval: transcription regulator; vertical-oval: transmembrane receptor; diamond: enzyme; square: cytokine; vertical-rectangle: G-protein coupled receptor; broken-lined vertical-rectangle: ion channel; and horizontal-diamond: peptidase. Marker color key—orange: predicted activation, blue: predicted inhibition. Connecting line color key—orange: activation; blue: inhibition; yellow: findings are inconsistent with the state of the downstream molecule; and gray: not predicted. High resolution versions of these networks are provided in the Supplementary Figure S2.

**Figure 6:**
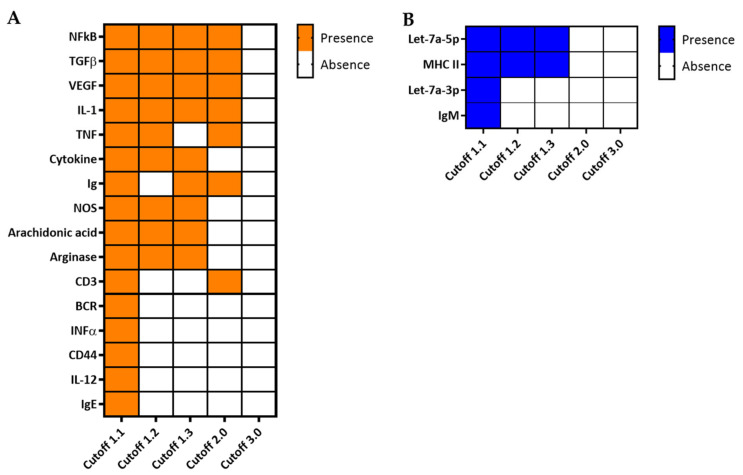
Key features identified in molecular networks relevant to immune/inflammatory processes in high-risk T1D subjects and the impact of the fold-change cutoff value on their identification. Comparison analysis in IPA software was applied to individual networks with scores at 10 or above, and key molecules were inferred based on curated integrated molecular networks generated at the indicated cutoff values (see Supplementary Figure S3). (**A**) Key molecules predicted to be activated in high-risk T1D subjects compared to healthy controls. Most prominent features, such as NF-κB, TGF-β, VEGF, IL-1, and TNF, were consistently identified in networks predicted at all cutoff values ≤2.0; none were yielded at cutoff 3.0. (**B**) Key molecules that were predicted to be negatively regulated (inhibited) also decreased with increasing the threshold cutoff values. Orange color indicates features predicted as activated and blue color as inhibited.

Finally, to demonstrate the potential of integrating parallel multi-omics in the identification of biomarker signature(s) for T1D, we used the “Integrated Molecular Network” analysis module in IPA to predict networks based on the combined quadra-omics dataset. We also subjected the dataset to the various feature selection stringencies (cutoff values) to evaluate the impact of the thresholding strategy on this type of analysis as well. The analyses yielded several molecular networks based on the integrated quadra-omics dataset with the different cutoff values. Each predicted network was assigned a score by IPA, and only those with a score of 10 or above were further considered in the detailed analyses (a network score is defined as the −log[*p*-value]; https://go.qiagen.com/IPA-transcriptomics-whitepaper, accessed on 16 February 2021). We also evaluated the predicted networks for information input from the four omics datasets as a function of the cutoff value. The analyses showed that only a single network that included features from all four omics datasets was consistently predicted when using the cutoff values of 1.1, 1.2, and 1.3 (Figure 5 and Supplementary Figure S2). The contribution of the individual omics datasets in these integrative analyses is highlighted in Figure 5B–E. Notably, at cutoff 2.0, there was no contribution by lipidomics to the analysis (see Table 1 and Figure 1) and the complexity of predicted networks relevant to immune/inflammatory functions was reduced. At cutoff 3.0, no predictions of any networks could be made in the current dataset from 4 + 4 subjects. Interestingly, molecules/features identified by the integrative analyses in the quadra-omics dataset at the lower cutoff values consistently corresponded to essential nodes in signaling pathways involved in the activation of immune/inflammatory reactions. They included the nuclear factor kappa B (NF-κB), transforming growth factor beta (TGF-β), vascular endothelial growth factor (VEGF), arachidonic acid, and arginase—all predicted to be activated (Figure 5A). Consistently, members of the Let-7 family of microRNAs, which are negative regulators of inflammation, were predicted to be inhibited (Figure 5A). Further key molecules within the predicted networks that were found as a function of the cutoff value to be upregulated or inhibited are shown in Figure 6A,B, respectively (also see Supplementary Figure S3). Notably, the consistent yields of immune-related outcomes by this integrative analysis approach with the lower cutoff values highlighted the importance of the thresholding strategy in identifying T1D relevant molecules, and the potential of such molecules in providing integrated T1D biomarker signature(s) for diagnostic and prognostic purposes. 

## 4. Discussion

We performed parallel untargeted multi-omics to obtain a comprehensive picture of perturbations in the proteome, metabolome, lipidome, and transcriptome in the blood of human subjects considered at a high risk for developing T1D compared to healthy controls. We also implemented an integrative analysis approach in the combined quadra-omics dataset and evaluated the impact of the thresholding strategy in the feature selection on the analytical outcomes. This work exploited the recent advances in analytical techniques that now allow the use of small-size samples in parallel multi-omics analyses and employed existing and newly developed network-focused computational tools to highlight the potential of parallel multi-omics in extracting mechanistic insight into T1D pathogenesis and identifying associated candidate biomarker signatures. These proof-of-concept studies were performed on a limited number of subjects (*n* = 4 + 4) but they, nevertheless, represent a first step toward (a) identifying novel and reliable early biomarker signatures in pre-diabetic subjects at various risk levels for T1D; (b) conveying unique information on the immunopathology of T1D by highlighting metabolic/signaling pathways that are most affected early in T1D and during progression toward its onset and clinical diagnosis; (c) facilitating the development of new diagnostic and prognostic tools; and d) accelerating the discovery/development of novel preventive and therapeutic strategies.

Technical advances in omics approaches (e.g., LC-MS/MS) now allow the detection, identification, and quantification of hundreds and even thousands of features in very small sample volumes. For example, these allowed us to complete untargeted metabolomics studies with human islets exposed to different stressors [79] as well as with male and female NOD mice that either did or did not progress to diabetes [80]. Even more notably, we were recently able to quantify hundreds of metabolites and proteins in longitudinal plasma and aqueous humor samples as small as 5 μL [61,81]. Moreover, advances in computational tools and software now make possible complex pathway-specific readouts based on data from multiple platforms to provide insight into comprehensive changes in the proteome, metabolome, lipidome, and transcriptome in the context of a given disease. Here, we used such tools in the *Ingenuity Pathway Analysis* (IPA) software package, which enabled analyses in integrated datasets obtained from four parallel omics-type studies in the context of T1D. Ultimately, in line with other general multi-omics approaches [55,56], the goal of our strategy using parallel multi-omics is to reveal network-based multi-signal interactions to construct novel and clinically relevant integrated biomarker signatures in association with T1D progression from the earliest pathogenic stages until the manifestation of overt symptoms at clinical diagnosis (i.e., dysglycaemia, glucose intolerance, hyperglycemia, and glycosuria).

In the present data obtained in blood samples from four high-risk T1D subjects and four healthy controls, individual omics analyses were able to independently identify a few significantly altered features in the high-risk group. For instance, our lipidomics studies indicated significantly increased LPC(18:1) in the high-risk T1D subjects, which is consistent with previous observations by others suggesting altered lipid levels as a possible early T1D biomarker. For example, in the BABYDIAB study involving 1,650 children of parents with T1D, Pflueger and co-workers found significantly higher levels of a lipid cluster dominated by polyunsaturated fatty acid (PUFA)-containing phosphatidylcholines (and triglycerides) [43]. We also previously found several phosphocholine derivatives, such as 1-palmitoyl-2-oleoyl-GPC (glycero-3-phosphocholine), 1-palmitoyl-2-arachidonoyl-GPC, and 1-stearoyl-2-arachidonoyl-GPC, as significantly increased early in pre-diabetic NOD mice that later progressed to diabetes [80]. On the other hand, Oresic and co-workers found lower levels of phosphatidylcholines in children who later progressed to T1D in early childhood (DIPP study of 129 genetically high-risk children) [52]. Moreover, our metabolomics studies here identified a statistically significant decrease in alanine and an increase in pyruvic acid (pyruvate) in the high-risk T1D subjects. Both metabolites are intimately connected to T1D [82,83,84]. The increase in pyruvate is a component of the Warburg effect, where increased pyruvate levels result from an inflammation-induced metabolic shift that stops its processing through the TCA cycle and, whereby, increasing the glycolysis pathway even at sufficient oxygen tensions [85,86]. In diabetes, there is a metabolic shift towards anaerobic glucose metabolism and an interruption of oxidative phosphorylation and the excess of pyruvate is diverted toward the production of lactate [87]. Alanine levels were also shown to be reduced in several tissues in recent studies in an experimental model of streptozotocin-induced diabetes [88]. However, the reduction in alanine levels is not inconsequential, as T1D-associated severe hypoglycemia can be corrected by alanine administration [83]. Moreover, alanine is an activator of the AMP kinase pathway which is essential in lowering glycemia [89]. Therefore, our findings showing reduced plasma levels of alanine and increased pyruvate are consistent with the metabolic shift observed in T1D. Furthermore, the current parallel transcriptomics studies showed significant increases in the levels of several plasma microRNAs (miR-182-5p, miR-486-5p, and miR-3605-5p) (Figure 2). Two of these miRNAs, miR182-5p and miR486-5p, were reported to be significantly increased in children with recent T1D diagnosis [90,91], and miR-486-5p has been suggested as a possible biomarker of diabetes risk in children [92,93]. However, to the best of our knowledge, alternations in plasma levels of miR3605-5p have not been previously highlighted in subjects at risk for developing T1D. Surprisingly, these independent analyses based on fold-change alone did not identify any proteins with significant differences between the high-risk T1D group and controls, likely due to the fold-change compression associated with the tandem mass tagging method we used to barcode the samples [72]. Nonetheless, the general agreement of our current preliminary findings with prior studies warrants additional parallel multi-omics studies in larger cohorts for further validation and the discovery of reliable T1D biomarker signatures.

While such analyses in the individual omics datasets pinpointed some relevant early indicators of increased inflammation and immune activation, the main strength of these parallel multi-omics studies comes from their integration and combined analysis. We also found that the thresholding strategy in the feature selection is critical, as it considerably impacted the quantity and quality of the information yields in a T1D-relevant context (Figure 3 and Figure 6). Increasing the stringency of feature selection (i.e., increasing the fold-change cutoff value) could lower the noise in the input data and, hence, the false discovery rate, whereby, strengthening the power of the prediction; however, it also results in decreasing the number of predicted functions, pathways, and networks particularly hampering evaluation of smaller biological datasets, where access to samples could be inherently limited by the nature of the disease, its prevalence, and the affected population. Thus, thresholding considerations should be applied carefully in these analyses, which is why we performed here the detailed analyses at different stringency levels. Notably, predictions of the same functions, pathways, and networks were consistently made at several, less stringent, cutoff values in our current dataset. We recently showed that the prediction accuracy in a small dataset was higher based on complete peak patterns in whole electropherograms rather than a few discriminative peaks selected based on significance, despite the potentially higher noise (non-significantly different peaks) in the compared electropherograms [94]. For instance, inflammatory response, proliferation of immune cells including T-lymphocytes, especially CD4+, and their increased motility, and the activation of macrophages and ROS generation were only indicated at the three lower cutoff values (Figure 3). The increased motility is consistent with our previous findings in animal models showing increased displacement and velocity of CD4+ and CD8+ T-lymphocytes in association with the onset of islet rejection and autoimmune T1D [95,96,97]. Similarly, pro-inflammatory factors such as NF-κB, TGF-β, VEGF, IL-1, and arachidonic acid were also consistently shown to be activated/upregulated at cutoff values ≤2.0 in the current analyses. Moreover, among the various detected microRNAs, the let-7 family which is conserved across different species [98], stood out in the prediction as distinctly inhibited in the high-risk T1D subjects compared to healthy controls (Figure 5C, Figure S2B and Table 2, Table S2). Interestingly, mounting evidence in recent years points to a possible role of some of these miRNAs in suppressing inflammation and the immune response [99,100,101,102]. Notably, the IPA-predicted network based on the current data showed direct inhibition of let-7a-5p consequent of the activation of both TGF-β and MAP2K1/2 (Figure 5A). This finding is consistent with a recent report in children with T1D [103]. Moreover, alterations in arginase plasma levels were also shown in the integrative analysis. Arginase plays an essential role in immune cell polarization, particularly, in macrophages toward a pro-inflammatory phenotype and ROS production [104,105,106]. Overall, the current findings highlight a molecular profile consistent with a pro-inflammatory milieu, which further underscores the potential of parallel multi-omics in yielding mechanistic insight in association with the disease-risk in subjects with various risk levels for developing T1D. While these are preliminary proof-of-concept results in a limited number of subjects and despite the possibility of potential differences in association with gender, age, and ethnicity, they highlight the potential of identifying in larger and longitudinal cohorts novel biomarker signatures composed of key proteins, metabolites, lipids, and RNA species that may prove valuable as early diagnostic and prognostic tools in at-risk T1D subjects and during their progression toward clinical diagnosis.

Finally, despite the limited number of samples obtained from the high-risk T1D subjects at a single time point during their disease progression, the integrative analyses identified a candidate corresponding integrated biomarker signature in these subjects (Table 2). Notably, several of these miRNAs, including let-7a-5p, have been associated with T1D [32,105,106]. Moreover, although one of the samples in the high-risk T1D group was from a newly diagnosed subject, this likely did not influence the analyses since we did not observe significant variance in any of the identified analytes/features across the samples within this group (see Supplementary Figure S4). Candidate biomarkers were identified here using the biomarker prediction module of IPA software in each omics dataset (with feature selection at the 1.1 cutoff value) independently, as such prediction in the combined quadra-omics dataset is not currently possible due to limitations in IPA (see Supplementary Table S3 for a complete list of IPA-predicted candidate biomarkers). However, we anticipate the predictions of such integrated biomarker signatures to improve to a more manageable number of features with increasing the sample size and improved software capabilities in working with combined multi-omics datasets. Identification of signatures for specific disease stages may also become possible in multi-omics datasets obtained in longitudinal samples from the same at-risk subjects prior to and leading up to their T1D diagnosis. Nevertheless, careful examination of all the biomarker candidates for direct involvement in central nodes within the global molecular network—predicted in the integrative analysis of the combined quadra-omics dataset (Figure 5)—yielded the candidate integrated biomarker signature composed of several miRNAs plus metabolic and lipid features shown in Table 2; namely, miRNAs let-7a-5p, miR-130a-3p, miR-16-5p, miR-296-5p, miR-30c-5p, miR-324-3p, miR-532-5p, and miR-92a-3p plus uric acid and LPC 1-18:1(11Z). 

Notably, this biomarker signature did not include any protein features despite the high number of proteins identified at the cutoff value of 1.1 (Table 1 and Figure 1). It should also be noted that the contribution of lipidomics to the integrative analysis, and consequently to the predicted biomarkers and candidate biomarker signature, was somewhat limited in the present studies due to existing limitations inherent to the ‘translation’ of the lipid annotations recognized by the IPA software. In addition, measurements of lipid families identified in the lipidomics dataset with a single annotation (e.g., ceramides or phosphatidyl inositols) without the specific annotation of the individual lipid subspecies of those families, prevented their inclusion in the analyses. Therefore, the overall lipidomics contribution to the integrative analysis was likely underrepresented in this limited sample size dataset (Figure 1, Figure 5E). Curiously, leaving out the lipidomics dataset increased the coverage of predicted networks, which further suggests the importance of lipids in the molecular signaling underlying the involved immunological processes considered by the algorithms used in IPA. The analysis using three integrated datasets from the proteomics, metabolomics, and transcriptomics alone yielded additional immune/inflammatory molecular networks likely to be involved in T1D pathogenesis (see Supplementary Figure S3). 

Consistent with the above findings, the number of pathways and the complexity of predicted networks based on tri-omics increased with lowering the stringency of the feature selection. At cutoff values of 1.2 and 1.3, only a single network was identified for each, while two networks were returned at the cutoff value of 1.1 (Supplementary Figure S2). This, together with the current inability to make biomarker predictions in the integrated multi-omics dataset, further emphasize the need for (a) additional studies in larger cohorts, (b) establishing proper guidelines for feature selection strategies in such analyses to prevent overlooking relevant and possibly important information, and (c) new bioinformatics tools that can integrate data from multiple platforms in multi-omics studies for various analyses and information extraction, including prediction of integrated biomarker signatures. With the constant advancements in artificial intelligence and machine learning approaches, perhaps a framework for large-scale multiple imputation approaches and optimization-based high-dimensional pattern discovery can prove useful for biomarker identification in multi-omics datasets. For example, a multiple imputation approach repeating currently used one-go deterministic pattern discovery processes thousands or millions of times, with stochastic mechanisms appended to individual processes, might amplify the pattern discovery power of existing and new algorithms, including those in IPA, with the potential of solving demanding and fundamental challenges in data synchronization/integration.

## 5. Conclusions

In conclusion, our proof-of-concept parallel quadra-omics analyses consistently indicated increased activation, proliferation, and migration of immune cells, particularly CD4+ T cells and macrophages, in subjects at high risk of developing T1D. Integrated molecular network predictions highlighted central involvement and activation of NF-κB, TGF-β, VEGF, arachidonic acid, and arginase, as well as the inhibition of Let-7a-5p. The thresholding strategy in feature selection had a strong effect on the number of functions, pathways, and signaling networks identified in relation to T1D pathogenesis, but several were consistently found at multiple, less stringent cutoff values, despite the limited sample size in the current dataset. This highlights a major advantage of parallel multi-omics by increasing the data input in the integrative analyses while simultaneously compensating for the potentially increased noise in the data and improving the reliability of prediction capabilities of newer software tools such as IPA even at lower cutoffs values [110,111]. The current studies also highlighted existing limitations in the inconsistent annotation methods across different omics approaches that hindered the integrative analysis and associated biomarker prediction capabilities of the IPA software, underscoring the need for expanded computational approaches and bioinformatics toolkits to address these limitations. Nevertheless, the current proof-of-concept studies demonstrated the feasibility of parallel multi-omics in human blood samples and highlighted their utility in gaining conceptual insight into the complex pathogenic mechanisms of T1D, and their potential in establishing associated novel integrated biomarker signatures composed of protein, metabolic, lipid, and genetic features. With further development and longitudinal data from larger cohorts, parallel multi-omics may ultimately lead to the definitive biomarkers signatures that make possible the early classification of progressors and non-progressors among subjects considered at risk for developing T1D.

## Figures and Tables

**Table 1:** Summary of feature numbers in each omics dataset as a function o.f cutoff value. Total numbers of features/molecules identified in all samples (without exception) obtained from high-risk T1D and healthy subjects (*n* = 4 each), and numbers of remaining features after data selection using different fold-change cutoff values.

Fold-Change ^1^	Proteomics	Transcriptomics	Metabolomics	Lipidomics	Total
All	2292	328	75	41	2736
1.1	260	95	75	8	438
1.2	151	87	75	5	318
1.3	107	80	75	3	265
2.0	18	47	30	0	95
3.0	7	29	0	0	36

^1^ Used as cutoff value for feature selection in all the datasets and input into the integrative analysis in *IPA* software (QIAGEN Bioinformatics).

**Table 2:** A potential integrated biomarker signature in high-risk T1D subjects. Features were selected from those predicted by IPA in each omics dataset based on their involvement in central nodes within the global molecular network generated from the combined quadra-omics dataset at cutoff value of 1.1.

	Expression			
Feature	Fold-Change ^1^	*p*-Value ^2^	IPA-Predicted Function/Disorder	Link to T1D
let-7a-5p	1.834	0.133	Inflammation of organ	
miR-130a-3p	1.503	0.745	Chronic inflammatory disorder	
miR-16-5p	2.954	0.151	Maturation of monocytes, chronic inflammatory disorder, inflammation of organ	[107]
miR-296-5p	1.444	0.696	Inflammation of organ	[108]
miR-30c-5p	1.39	0.326	Chronic inflammatory disorder, inflammation of organ	
miR-324-3p	2.682	0.468	Inflammation of organ	[90]
miR-532-5p	2.484	0.122	Chronic inflammatory disorder, inflammation of organ	
miR-92a-3p	21.029	0.146	Induction of follicular T helper precursor cells, chronic inflammatory disorder, inflammation of organ	
Uric acid	2.008	0.872	Activation of macrophages, immune response of cells, induction of peripheral blood lymphocytes, inflammation of organ	[109]
LPC 1-18:1(11Z)	1.549	0.00949	None	

^1^ Predicted expression fold-change in high-risk T1D subjects relative to healthy controls. ^2^ Non-adjusted *p*-value as obtained from IPA software based on comparisons of high-risk T1D subjects vs. healthy controls in each omics-type dataset independently (*t*-test); only LPC from the lipidomics dataset had *p* < 0.05.

## Data Availability

The multi-omics datasets generated and/or analyzed in the current study have been deposited in the following publicly accessible repositories: the NIH Common Fund’s National Metabolomics Data Repository (NMDR; www.metabolomicsworkbench.org, accessed on 16 February 2021) [112], accession #s ST001690 (doi:10.21228/M8B123) for the metabolomics dataset and ST001642 (doi:10.21228/M8ZX18) for the lipidomics dataset; the PRIDE database of ProteomeXchange (https://www.ebi.ac.uk/pride/, accessed on 16 February 2021), accession # PXD023541 for the proteomics dataset; and the Harvard Dataverse repository (doi.org/10.7910/DVN/A2OU24) for the transcriptomics dataset.

## Supplementary Materials

The following supplementary data are available online at https://www.mdpi.com/2218-273X/11/3/383/s1: Figure S1: Regulator Effects three-tier diagrams (networks) relevant to immune activation and inflammatory processes predicted at different analysis cutoff values; Figure S2: High resolution versions of the networks shown in Figure 5; Figure S3: Networks predicted at various levels of analysis stringency summarized in Figure 6; Figure S4: Scatter plot of *p*-values comparing the variance within the high-risk T1D group with and without the one new-onset subject. Table S1: Demographic, serological and other information of the subjects from whom the blood samples were obtained for this study; Table S2: Transcriptomics data quality control information; and Table S3: Complete list of candidate biomarkers predicted by IPA in the individual omics datasets without any curation in the context of molecular networks predicted in the integrated quadra-omics dataset.
