# Supplementary material for: Parallel Multi-Omics in High-Risk Subjects for the Identification of Integrated Biomarker Signatures of Type 1 Diabetes
Source: Biomolecules. 2021 Mar 4;11(3):383. doi: 10.3390/biom11030383 (PMC7999903; doi:10.3390/biom11030383)
Supplement: Supplementary file 1 [file biomolecules-11-00383-s001.pdf]

Supplementary Materials

Parallel Multi-Omics in High-Risk Subjects for the Identification of Integrated Biomarker Signatures of Type 1 Diabetes

Oscar Alcazar [1,#], Luis F. Hernandez [2,#], Ernesto S. Nakayasu [3], Carrie D. Nicora [4], Charles Ansong [5], Michael J. Muehlbauer [6], James R. Bain [7], Ciara J. Myer [8], Sanjoy K. Bhattacharya [9], Peter Buchwald [10,#,*], and Midhat H. Abdulreda [11,#,*]

1 University of Miami Miller School of Medicine, Diabetes Research Institute, Miami, FL, USA; email: o.alcazar@miami.edu

2 University of Miami Miller School of Medicine, Diabetes Research Institute, Miami, FL, USA; email: lfh34@miami.edu

3 Biological Sciences Division, Pacific Northwest National Laboratory, Richland, WA, USA; email: ernesto.nakayasu@pnnl.gov

4 Biological Sciences Division, Pacific Northwest National Laboratory, Richland, WA, USA; email: carrie.nicora@pnnl.gov

5 Biological Sciences Division, Pacific Northwest National Laboratory, Richland, WA, USA; email: Charles.Ansong@pnnl.gov

6 Duke Molecular Physiology Institute, Duke University Medical Center, Durham, NC, USA. email: michael.muehlbauer@duke.edu

7 Duke Molecular Physiology Institute, Duke University Medical Center, Durham, NC, USA. email: james.bain@duke.edu

8 University of Miami Miller School of Medicine, Department of Ophthalmology and Miami Integrative Metabolomics Research Center, Miami, FL, USA; email: cxm1496@miami.edu

9 University of Miami Miller School of Medicine, Department of Ophthalmology and Miami Integrative Metabolomics Research Center, Miami, FL, USA. email: sbhattacharya@miami.edu

10 University of Miami Miller School of Medicine, Diabetes Research Institute, and Department of Molecular and Cellular Pharmacology, Miami, FL, USA. email: pbuchwald@miami.edu

11 University of Miami Miller School of Medicine, Diabetes Research Institute, Department of Surgery, Department of Microbiology and Immunology, and Department of Ophthalmology, Miami, FL, USA; email: mabdulreda@miami.edu

# These authors contributed equally

* Correspondence emails mabdulreda@miami.edu or pbuchwald@miami.edu

Supplementary Materials Content

- **Figure S1.** Regulator Effects three-tier diagrams (networks) relevant to immune activation and inflammatory processes predicted at different analysis cutoff threshold values.

- **Figure S2**. High-resolution versions of the networks shown in **Figure 5**.

- **Figure S3.** Networks predicted at various levels of analysis stringency summarized in **Figure 6**.

- **Figure S4.** Scatter plot of *p*-values comparing (by t-test) the variance within the high-risk T1D group with and without the one new-onset subject.

- **Table S1.** Demographic, serological, and other information of subjects from whom blood samples were obtained in this study.

- **Table S2.** Transcriptomics data quality control information.

- **Table S3.** Complete list of candidate biomarkers predicted by *IPA* in the individual omics datasets without any curation in the context of the global molecular network predicted in the integrated multi-omics dataset at fold-change cutoff value of 1.1 during feature selection in the integrative analysis.

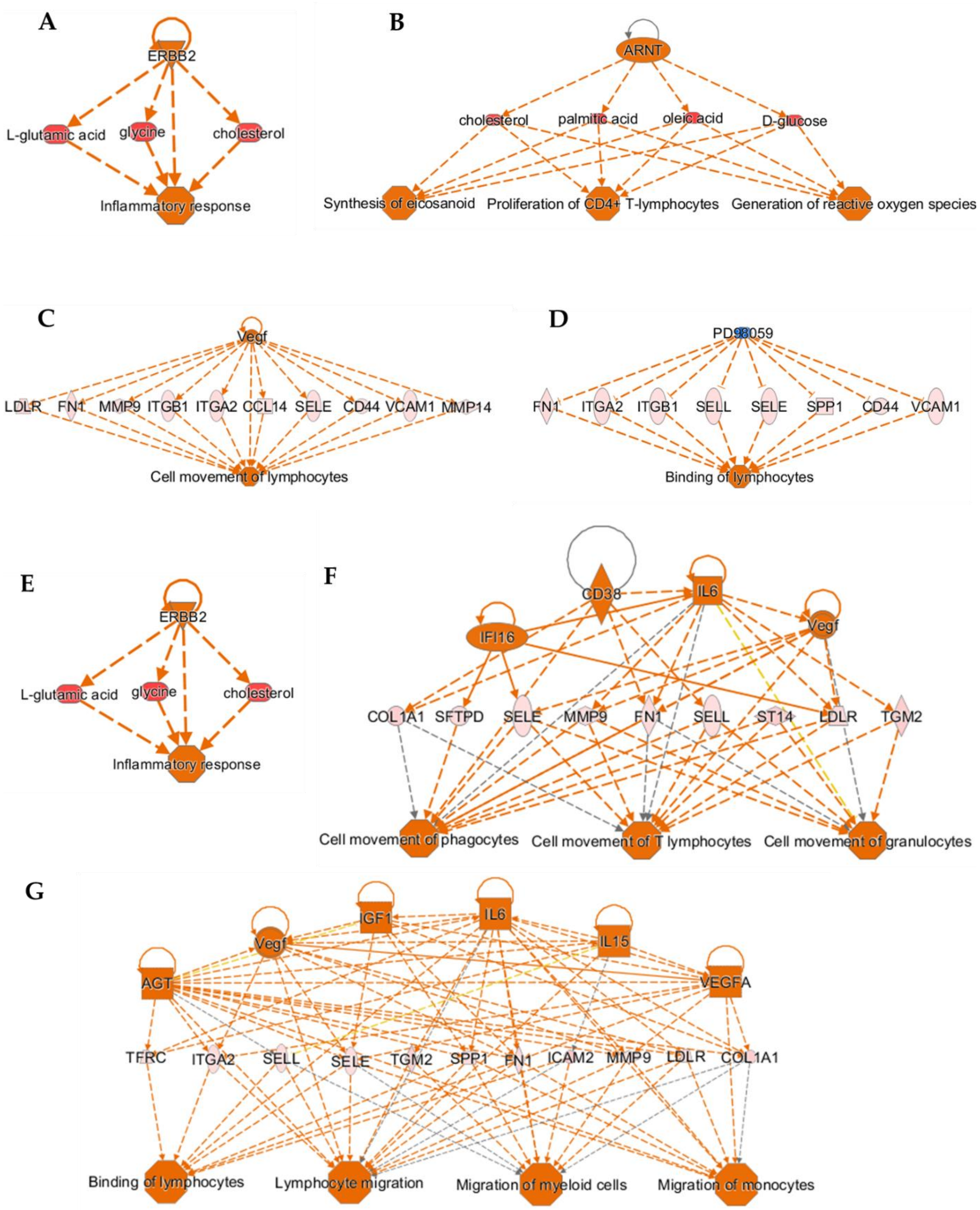
A
ERBB2
L-glutamic acid
glycine
cholesterol
Inflammatory response
B
ARNT
cholesterol
palmitic acid
oleic acid
D-glucose
Synthesis of eicosanoid
Proliferation of CD4+ T-lymphocytes
Generation of reactive oxygen species
C
Vegf
LDLR
FN1
MMP9
ITGB1
ITGA2
CCL14
SELE
CD44
VCAM1
MMP14
Cell movement of lymphocytes
D
PD98059
FN1
ITGA2
ITGB1
SELL
SELE
SPP1
CD44
VCAM1
Binding of lymphocytes
E
ERBB2
L-glutamic acid
glycine
cholesterol
Inflammatory response
F
CD38
IL6
IFI16
Vegf
COL1A1
SFTPD
SELE
MMP9
FN1
SELL
ST14
LDLR
TGM2
Cell movement of phagocytes
Cell movement of T lymphocytes
Cell movement of granulocytes
G
IGF1
IL6
Vegf
IL15
AGT
VEGFA
TFRC
ITGA2
SELL
SELE
TGM2
SPP1
FN1
ICAM2
MMP9
LDLR
COL1A1
Binding of lymphocytes
Lymphocyte migration
Migration of myeloid cells
Migration of monocytes

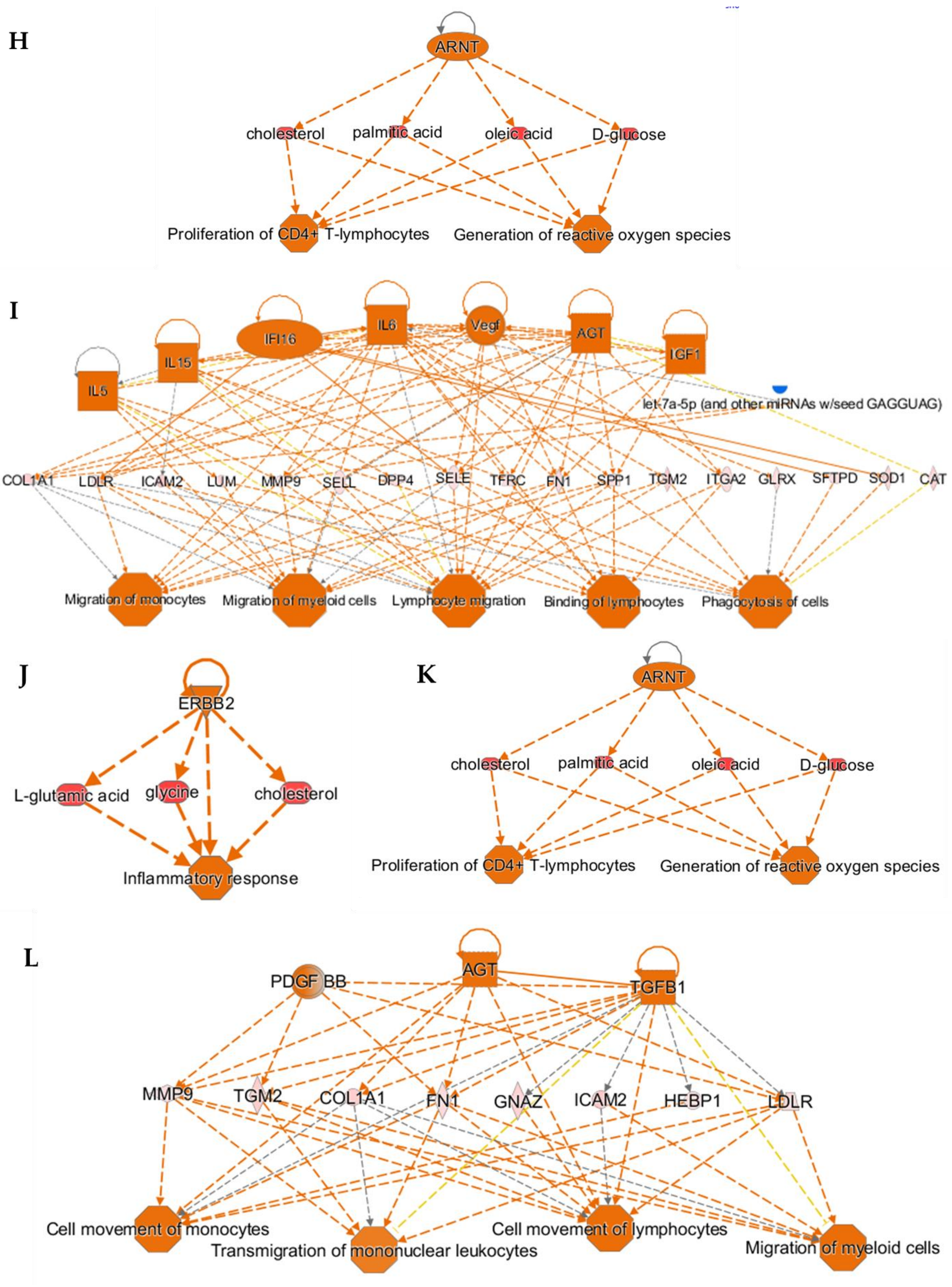

Figure S1 (corresponds to Figure 4.). Regulator Effects three-tier diagrams (networks) predicted at the different feature selection thresholds (fold-change cutoff values). Predictions at cutoff values **(A–D)** 1.1, **(E–I)** 1.2, and **(J–L)** 1.3 using the "Regulator Effects" module of the *IPA* software. Analyses at the cutoff values 2.0 or 3.0 did not yield any predictions. Marker key – Triangle: kinase horizontal-oval: transcription regulator; vertical-oval: transmembrane receptor; diamond: enzyme; square: cytokine; horizontal ellipse: metabolite; circle: other; octagon: function. Color key – orange: predicted activation, blue: predicted inhibition. Connecting line color key – orange: activation; blue: inhibition; yellow: findings are inconsistent with the state of the downstream molecule; and gray: not predicted.

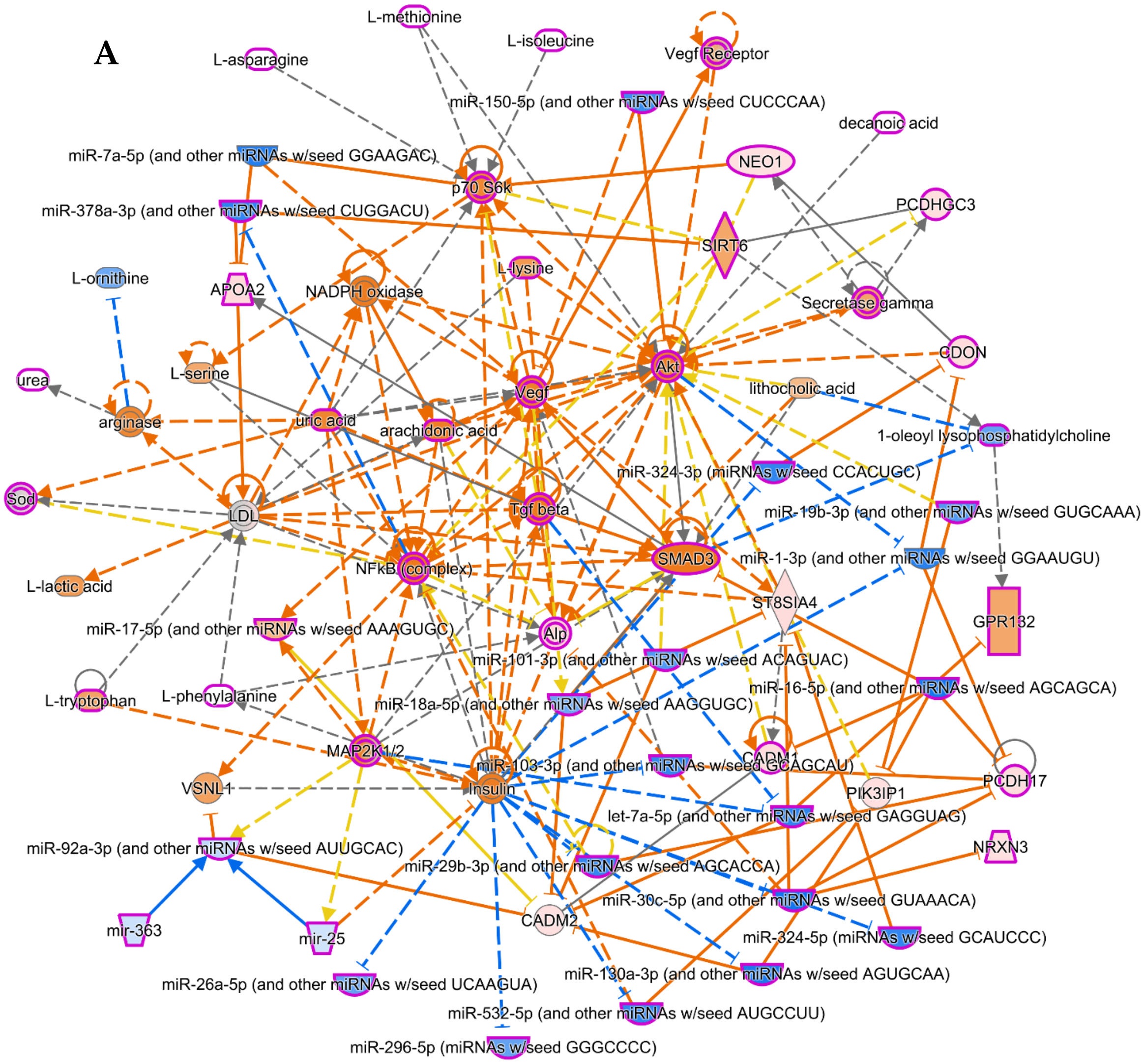
A
L-methionine
L-isoleucine
Vegf Receptor
L-asparagine
miR-150-5p (and other miRNAs w/seed CUCCCAA)
decanoic acid
miR-7a-5p (and other miRNAs w/seed GGAAGAC)
NEO1
p70 S6k
miR-378a-3p (and other miRNAs w/seed CUGGACU)
PCDHGC3
SIRT6
L-ornithine
L-lysine
APOA2
NADPH oxidase
Secretase gamma
urea
L-serine
Akt
CDON
Vegf
lithocholic acid
arginase
uric acid
arachidonic acid
1-oleoyl lysophosphatidylcholine
miR-324-3p (miRNAs w/seed CCACUGC)
Sod
LDL
Tgf beta
miR-19b-3p (and other miRNAs w/seed GUGCAAA)
SMAD3
miR-1-3p (and other miRNAs w/seed GGAAUGU)
NFkB (complex)
L-lactic acid
ST8SIA4
GPR132
miR-17-5p (and other miRNAs w/seed AAAGUGC)
Alp
miR-101-3p (and other miRNAs w/seed ACAGUAC)
miR-16-5p (and other miRNAs w/seed AGCAGCA)
L-tryptophan
L-phenylalanine
miR-18a-5p (and other miRNAs w/seed AAGGUGC)
MAP2K1/2
CADM1
miR-103-3p (and other miRNAs w/seed GCAGCAU)
VSNL1
Insulin
PIK3IP1
PCDH17
let-7a-5p (and other miRNAs w/seed GAGGUAG)
miR-92a-3p (and other miRNAs w/seed AUUGCAC)
NRXN3
miR-29b-3p (and other miRNAs w/seed AGCACCA)
miR-30c-5p (and other miRNAs w/seed GUAAACA)
mir-363
mir-25
CADM2
miR-324-5p (miRNAs w/seed GCAUCCC)
miR-130a-3p (and other miRNAs w/seed AGUGCAA)
miR-26a-5p (and other miRNAs w/seed UCAAGUA)
miR-532-5p (and other miRNAs w/seed AUGCCUU)
miR-296-5p (miRNAs w/seed GGGCCCC)

B

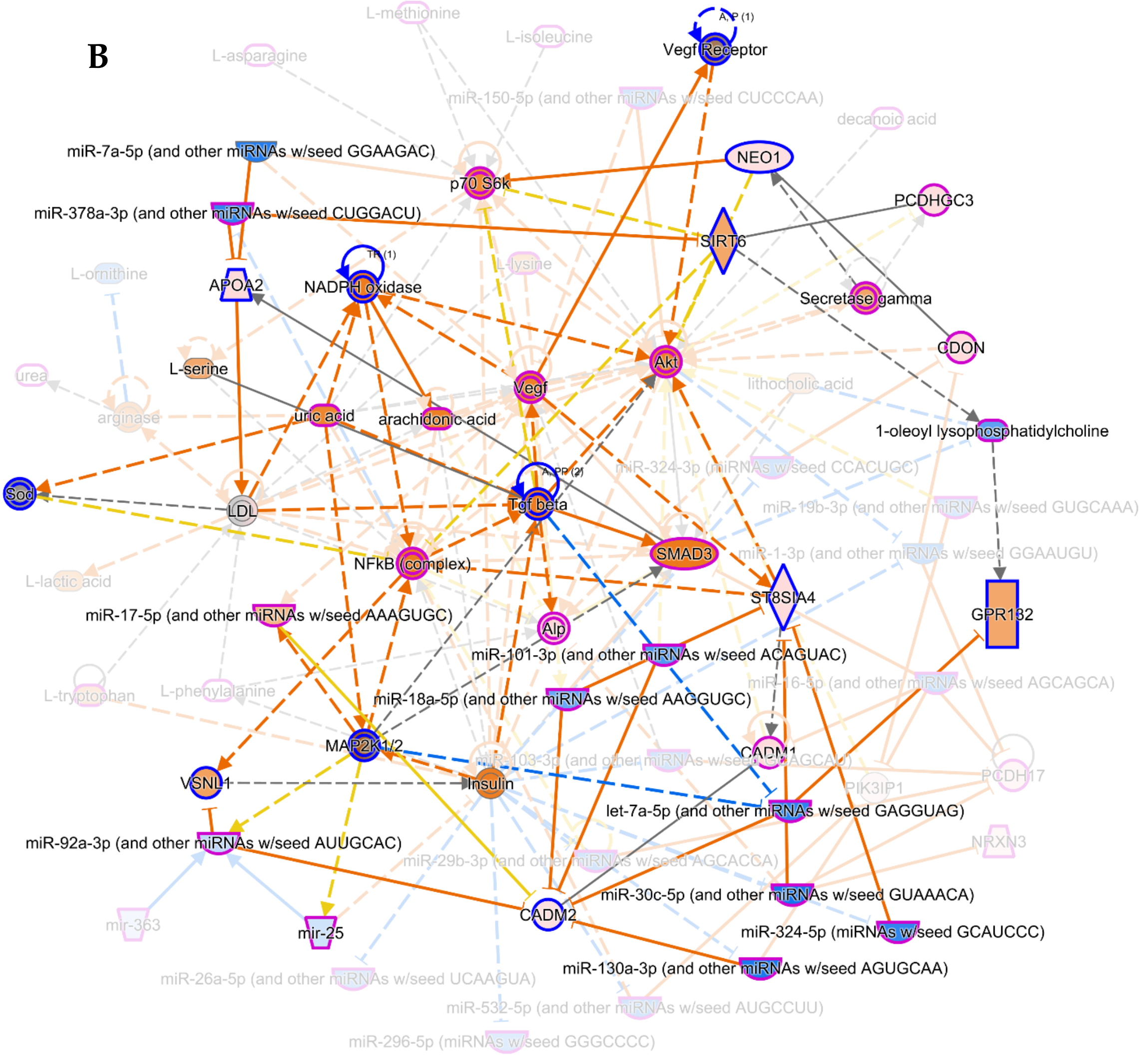

L-methionine
L-isoleucine
Vegf Receptor
L-asparagine
miR-150-5p (and other miRNAs w/seed CUCCCAA)
decanoic acid
miR-7a-5p (and other miRNAs w/seed GGAAGAC)
NEO1
p70 S6k
PCDHGC3
miR-378a-3p (and other miRNAs w/seed CUGGACU)
SIRT6
L-ornithine
L-lysine
APOA2
NADPH oxidase
Secretase gamma
CDON
urea
L-serine
Akt
Vegf
lithocholic acid
arginase
uric acid
arachidonic acid
1-oleoyl lysophosphatidylcholine
miR-324-3p (miRNAs w/seed CCACUGC)
Sod
LDL
Tgf beta
miR-19b-3p (and other miRNAs w/seed GUGCAAA)
SMAD3
miR-1-3p (and other miRNAs w/seed GGAAUGU)
NFkB (complex)
L-lactic acid
ST8SIA4
miR-17-5p (and other miRNAs w/seed AAAGUGC)
GPR182
Alp
miR-101-3p (and other miRNAs w/seed ACAGUAC)
miR-16-5p (and other miRNAs w/seed AGCAGCA)
L-tryptophan
L-phenylalanine
miR-18a-5p (and other miRNAs w/seed AAGGUGC)
MAP2K1/2
CADM1
miR-103-3p (and other miRNAs w/seed GCAGCAU)
PCDH17
VSNL1
Insulin
PIK3IP1
let-7a-5p (and other miRNAs w/seed GAGGUAG)
miR-92a-3p (and other miRNAs w/seed AUUGCAC)
NRXN3
miR-29b-3p (and other miRNAs w/seed AGCACCA)
miR-30c-5p (and other miRNAs w/seed GUAAACA)
mir-363
CADM2
mir-25
miR-324-5p (miRNAs w/seed GCAUCCC)
miR-130a-3p (and other miRNAs w/seed AGUGCAA)
miR-26a-5p (and other miRNAs w/seed UCAAGUA)
miR-532-5p (and other miRNAs w/seed AUGCCUU)
miR-296-5p (miRNAs w/seed GGGCCCC)

C

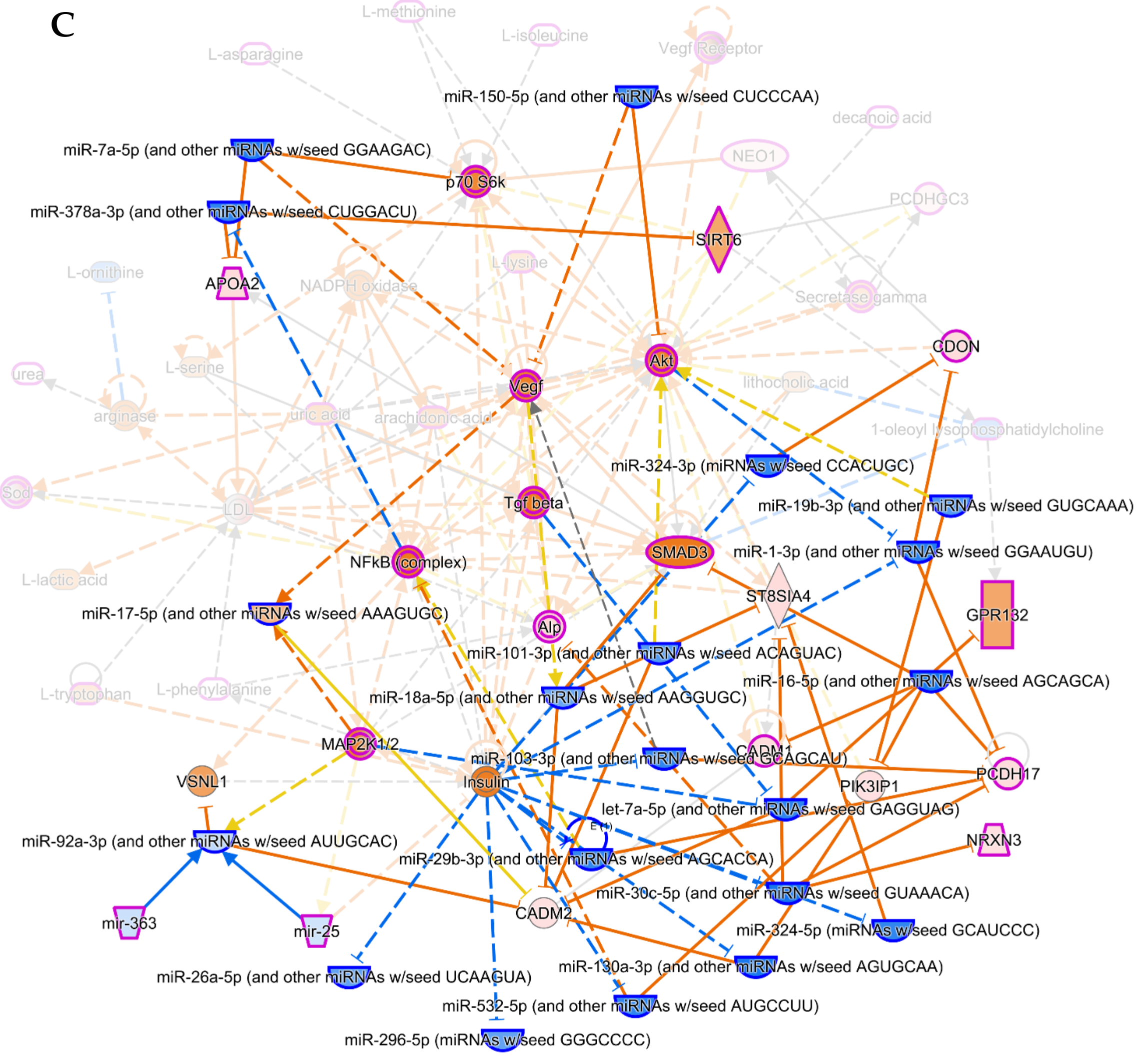

L-methionine
L-isoleucine
Vegf Receptor
L-asparagine
miR-150-5p (and other miRNAs w/seed CUCCCAA)
decanoic acid
miR-7a-5p (and other miRNAs w/seed GGAAGAC)
NEO1
p70 S6k
PCDHGC3
miR-378a-3p (and other miRNAs w/seed CUGGACU)
SIRT6
L-ornithine
L-lysine
APOA2
NADPH oxidase
Secretase gamma
CDON
Akt
L-serine
urea
Vegf
lithocholic acid
arginase
uric acid
arachidonic acid
1-oleoyl lysophosphatidylcholine
miR-324-3p (miRNAs w/seed CCACUGC)
Sod
LDL
Tgf beta
miR-19b-3p (and other miRNAs w/seed GUGCAAA)
SMAD3
miR-1-3p (and other miRNAs w/seed GGAAUGU)
NFkB (complex)
L-lactic acid
ST8SIA4
miR-17-5p (and other miRNAs w/seed AAAGUGC)
GPR132
Alp
miR-101-3p (and other miRNAs w/seed ACAGUAC)
miR-16-5p (and other miRNAs w/seed AGCAGCA)
L-tryptophan
L-phenylalanine
miR-18a-5p (and other miRNAs w/seed AAGGUGC)
MAP2K1/2
CADM1
miR-103-3p (and other miRNAs w/seed GCAGCAU)
VSNL1
Insulin
PIK3IP1
PCDH17
let-7a-5p (and other miRNAs w/seed GAGGUAG)
miR-92a-3p (and other miRNAs w/seed AUUGCAC)
NRXN3
miR-29b-3p (and other miRNAs w/seed AGCACCA)
miR-30c-5p (and other miRNAs w/seed GUAAACA)
mir-363
CADM2
mir-25
miR-324-5p (miRNAs w/seed GCAUCCC)
miR-130a-3p (and other miRNAs w/seed AGUGCAA)
miR-26a-5p (and other miRNAs w/seed UCAAGUA)
miR-532-5p (and other miRNAs w/seed AUGCCUU)
miR-296-5p (miRNAs w/seed GGGCCCC)

D

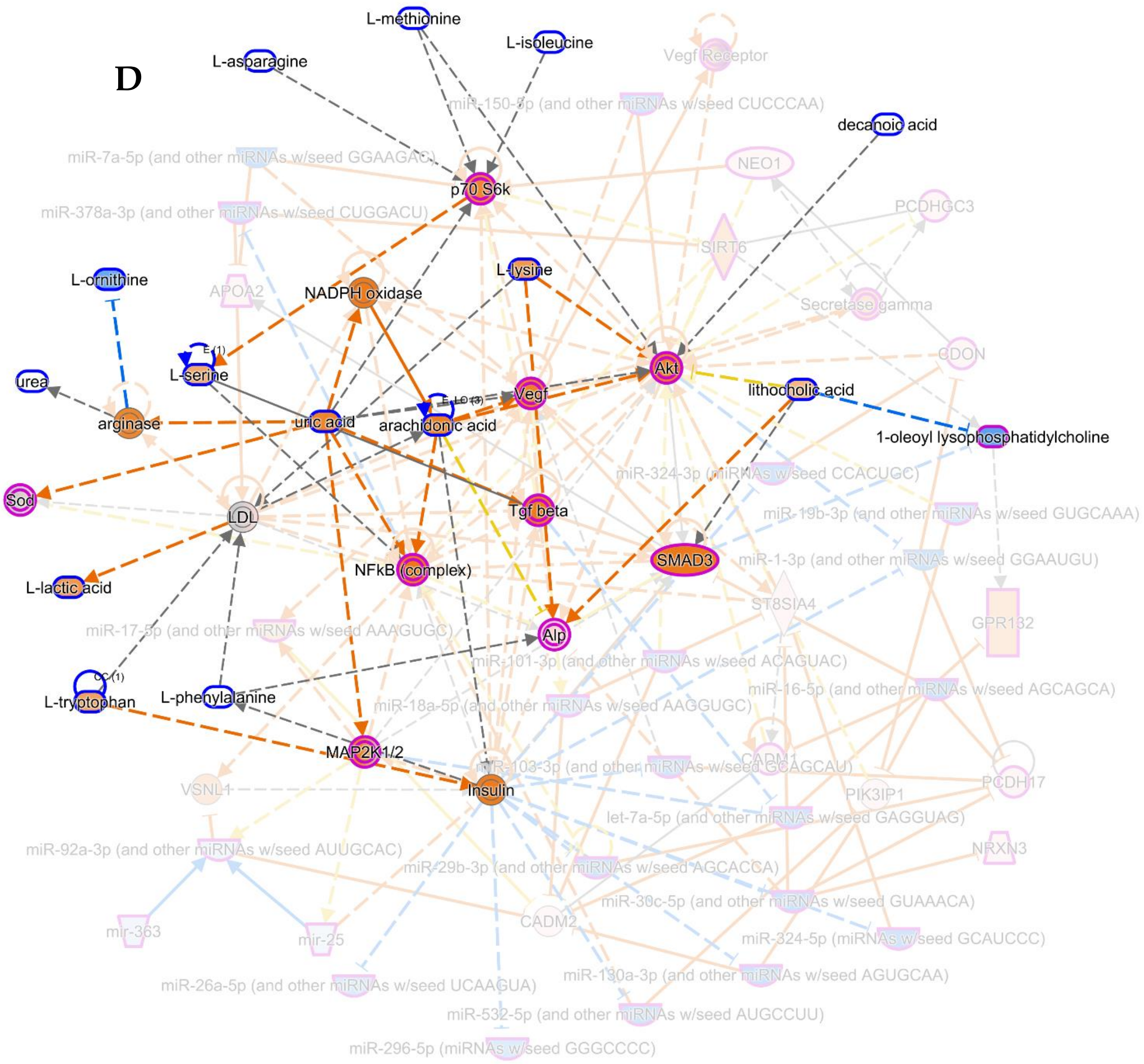
L-methionine
L-isoleucine
L-asparagine
Vegf Receptor
miR-150-5p (and other miRNAs w/seed CUCCCAA)
decanoic acid
miR-7a-5p (and other miRNAs w/seed GGAAGAC)
NEO1
p70 S6k
miR-378a-3p (and other miRNAs w/seed CUGGACU)
PCDHGC3
SIRT6
L-lysine
L-ornithine
APOA2
NADPH oxidase
Secretase gamma
CDON
L-serine
urea
Akt
Vegf
lithocholic acid
uric acid
arachidonic acid
arginase
1-oleoyl lysophosphatidylcholine
miR-324-3p (miRNAs w/seed CCACUGC)
Sod
LDL
Tgf beta
miR-19b-3p (and other miRNAs w/seed GUGCAAA)
SMAD3
miR-1-3p (and other miRNAs w/seed GGAAUGU)
L-lactic acid
NFkB (complex)
ST8SIA4
GPR182
miR-17-5p (and other miRNAs w/seed AAAGUGC)
Alp
miR-101-3p (and other miRNAs w/seed ACAGUAC)
miR-16-5p (and other miRNAs w/seed AGCAGCA)
L-tryptophan
L-phenylalanine
miR-18a-5p (and other miRNAs w/seed AAGGUGC)
MAP2K1/2
miR-103-3p (and other miRNAs w/seed GCAGCAU)
CADM1
PCDH17
VSNL1
Insulin
PIK3IP1
let-7a-5p (and other miRNAs w/seed GAGGUAG)
miR-92a-3p (and other miRNAs w/seed AUUGCAC)
NRXN3
miR-29b-3p (and other miRNAs w/seed AGCACCA)
miR-30c-5p (and other miRNAs w/seed GUAAACA)
CADM2
mir-363
mir-25
miR-324-5p (miRNAs w/seed GCAUCCC)
miR-130a-3p (and other miRNAs w/seed AGUGCAA)
miR-26a-5p (and other miRNAs w/seed UCAAGUA)
miR-532-5p (and other miRNAs w/seed AUGCCUU)
miR-296-5p (miRNAs w/seed GGGCCCC)

E

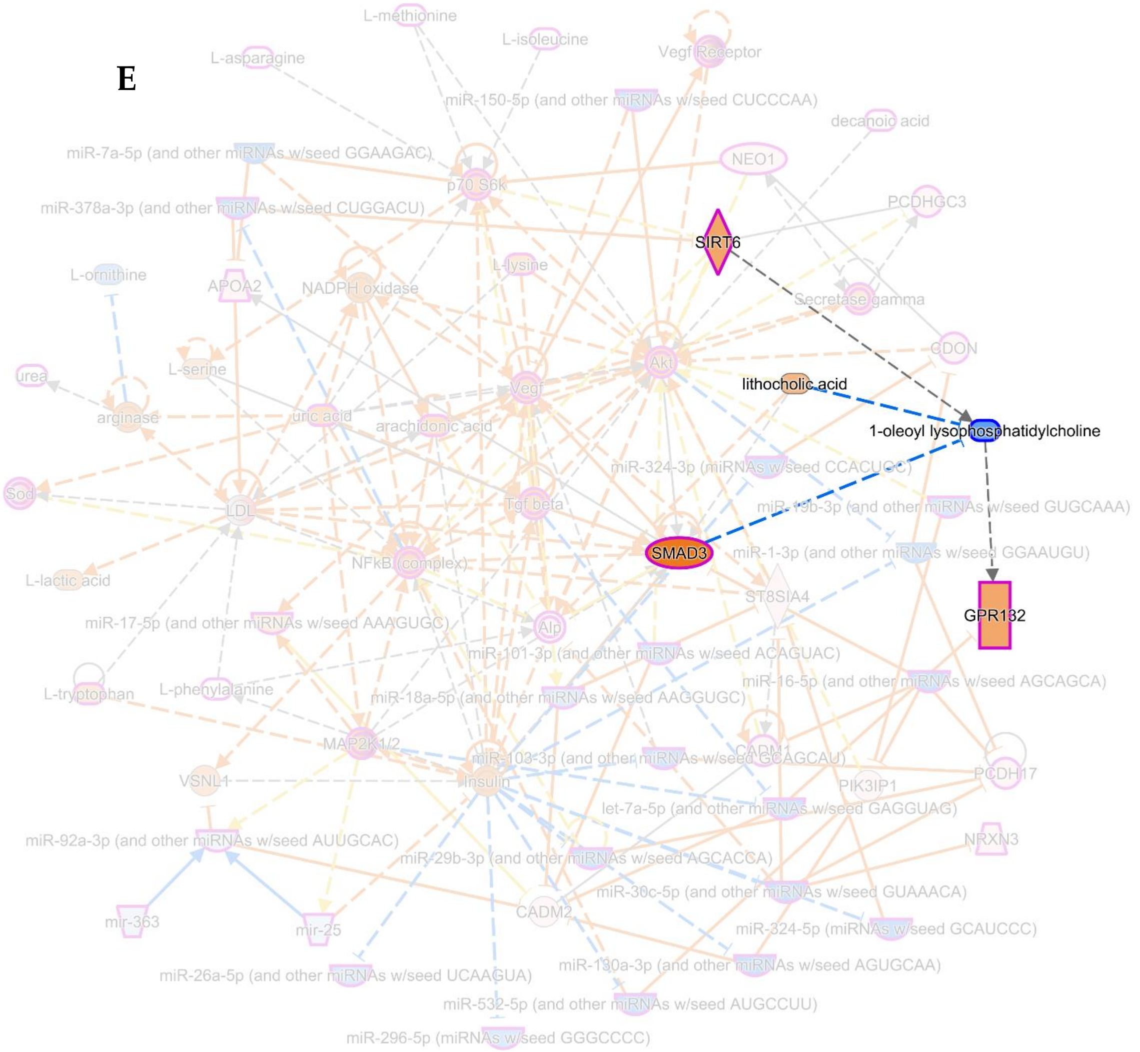

Figure S2 (corresponds to Figure 5). High-resolution versions of the networks shown in Figure 5. **(A)** The complete molecular network predicted in the integrated parallel quadra-omics dataset. **(B-E)** Zoomed parts of the network shown in **A** highlighting the contributions of **(B)** proteomics, **(C)** small-transcriptomics, **(D)** metabolomics, and **(E)** lipidomics. Marker shape key – horizontal-oval: transcription regulator; vertical-oval: transmembrane receptor; diamond: enzyme; square: cytokine; vertical-rectangle: G-protein coupled receptor; broken-lined vertical-rectangle: ion channel; horizontal-diamond: peptidase; trapezoid: transporter; concentric circle: complex; circle: other; horizontal ellipse: metabolite; cut ellipsoid: microRNA; and inverted trapezoid: microRNA family. Marker color key – orange: predicted activation, blue: predicted inhibition. Connecting line color key – orange: activation; blue: inhibition; yellow: findings are inconsistent with the state of the downstream molecule; and gray: not predicted.

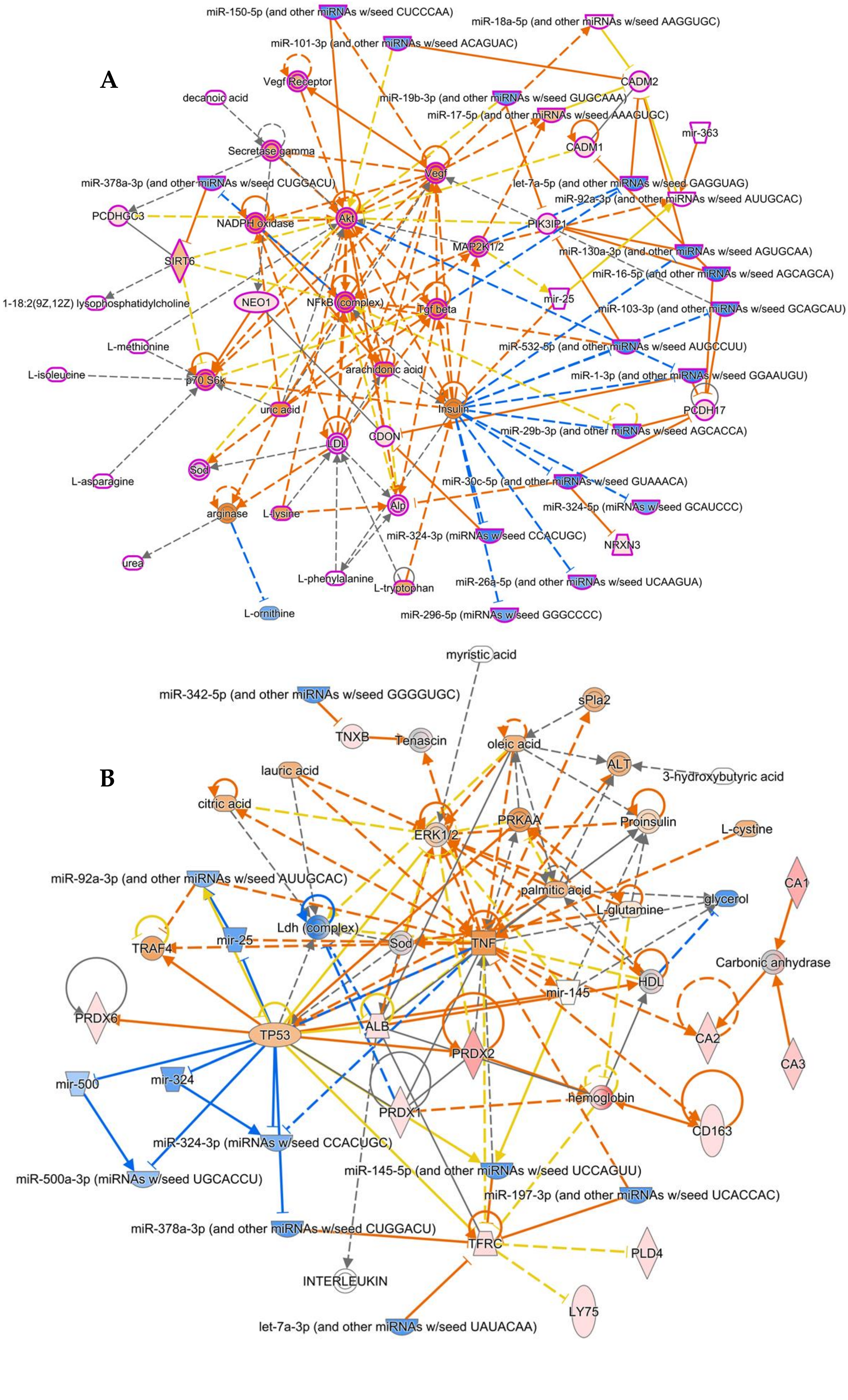
A
miR-150-5p (and other miRNAs w/seed CUCCCAA)
miR-18a-5p (and other miRNAs w/seed AAGGUGC)
miR-101-3p (and other miRNAs w/seed ACAGUAC)
Vegf Receptor
CADM2
decanoic acid
miR-19b-3p (and other miRNAs w/seed GUGCAAA)
miR-17-5p (and other miRNAs w/seed AAAGUGC)
mir-363
CADM1
Secretase gamma
Vegf
miR-378a-3p (and other miRNAs w/seed CUGGACU)
let-7a-5p (and other miRNAs w/seed GAGGUAG)
miR-92a-3p (and other miRNAs w/seed AUUGCAC)
PCDHGC3
NADPH oxidase
Akt
PIK3IP1
MAP2K1/2
miR-130a-3p (and other miRNAs w/seed AGUGCAA)
SIRT6
miR-16-5p (and other miRNAs w/seed AGCAGCA)
1-18:2(9Z,12Z) lysophosphatidylcholine
NEO1
NFkB (complex)
Tgf beta
mir-25
miR-103-3p (and other miRNAs w/seed GCAGCAU)
L-methionine
miR-532-5p (and other miRNAs w/seed AUGCCUU)
L-isoleucine
p70 S6k
arachidonic acid
miR-1-3p (and other miRNAs w/seed GGAAUGU)
uric acid
Insulin
PCDH17
LDL
CDON
miR-29b-3p (and other miRNAs w/seed AGCACCA)
Sod
L-asparagine
miR-30c-5p (and other miRNAs w/seed GUAAACA)
arginase
L-lysine
Alp
miR-324-5p (miRNAs w/seed GCAUCCC)
miR-324-3p (miRNAs w/seed CCACUGC)
NRXN3
urea
L-phenylalanine
L-tryptophan
miR-26a-5p (and other miRNAs w/seed UCAAGUA)
L-ornithine
miR-296-5p (miRNAs w/seed GGGCCCC)
B
myristic acid
miR-342-5p (and other miRNAs w/seed GGGGUGC)
sPla2
TNXB
Tenascin
oleic acid
lauric acid
ALT
3-hydroxybutyric acid
citric acid
ERK1/2
PRKAA
Proinsulin
L-cystine
CA1
miR-92a-3p (and other miRNAs w/seed AUUGCAC)
palmitic acid
glycerol
L-glutamine
Ldh (complex)
TRAF4
mir-25
Sod
TNF
Carbonic anhydrase
HDL
mir-145
PRDX6
ALB
CA2
TP53
CA3
PRDX2
mir-500
mir-324
hemoglobin
PRDX1
CD163
miR-324-3p (miRNAs w/seed CCACUGC)
miR-145-5p (and other miRNAs w/seed UCCAGUU)
miR-500a-3p (miRNAs w/seed UGCACCU)
miR-197-3p (and other miRNAs w/seed UCACCAC)
miR-378a-3p (and other miRNAs w/seed CUGGACU)
TFRC
PLD4
INTERLEUKIN
LY75
let-7a-3p (and other miRNAs w/seed UAUACAA)

C

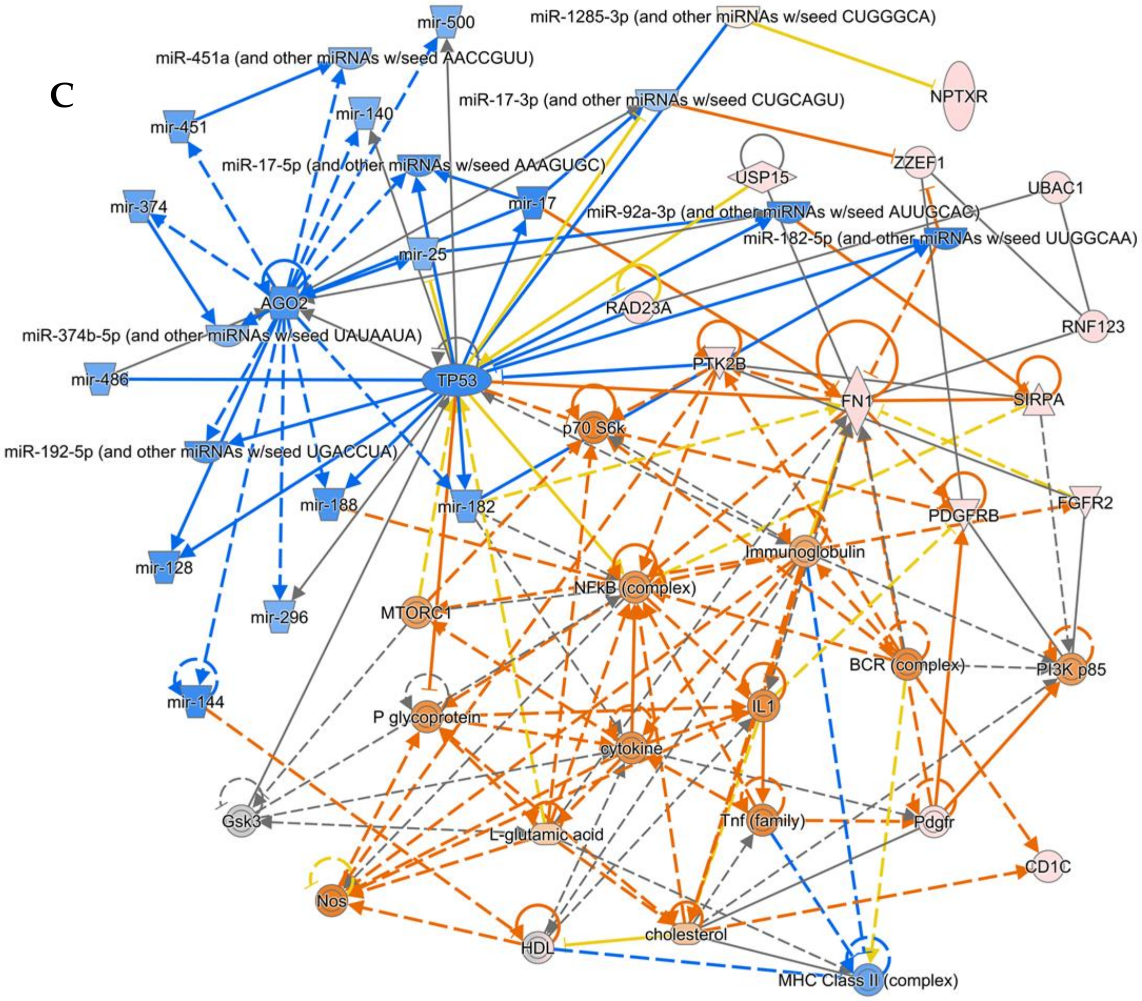
mir-500
miR-1285-3p (and other miRNAs w/seed CUGGGCA)
miR-451a (and other miRNAs w/seed AACCGUU)
NPTXR
miR-17-3p (and other miRNAs w/seed CUGCAGU)
mir-451
mir-140
ZZEF1
miR-17-5p (and other miRNAs w/seed AAAGUGC)
USP15
UBAC1
mir-374
mir-17
miR-92a-3p (and other miRNAs w/seed AUUGCAC)
miR-182-5p (and other miRNAs w/seed UUGGCAA)
mir-25
AGO2
RAD23A
miR-374b-5p (and other miRNAs w/seed UAUAAUA)
RNF123
PTK2B
mir-486
TP53
FN1
SIRPA
p70 S6k
miR-192-5p (and other miRNAs w/seed UGACCUA)
mir-188
mir-182
PDGFRB
FGFR2
Immunoglobulin
mir-128
NFkB (complex)
MTORC1
mir-296
BCR (complex)
PI3K p85
mir-144
IL1
P glycoprotein
cytokine
Gsk3
L-glutamic acid
Tnf (family)
Pdgfr
CD1C
Nos
cholesterol
HDL
MHC Class II (complex)

D

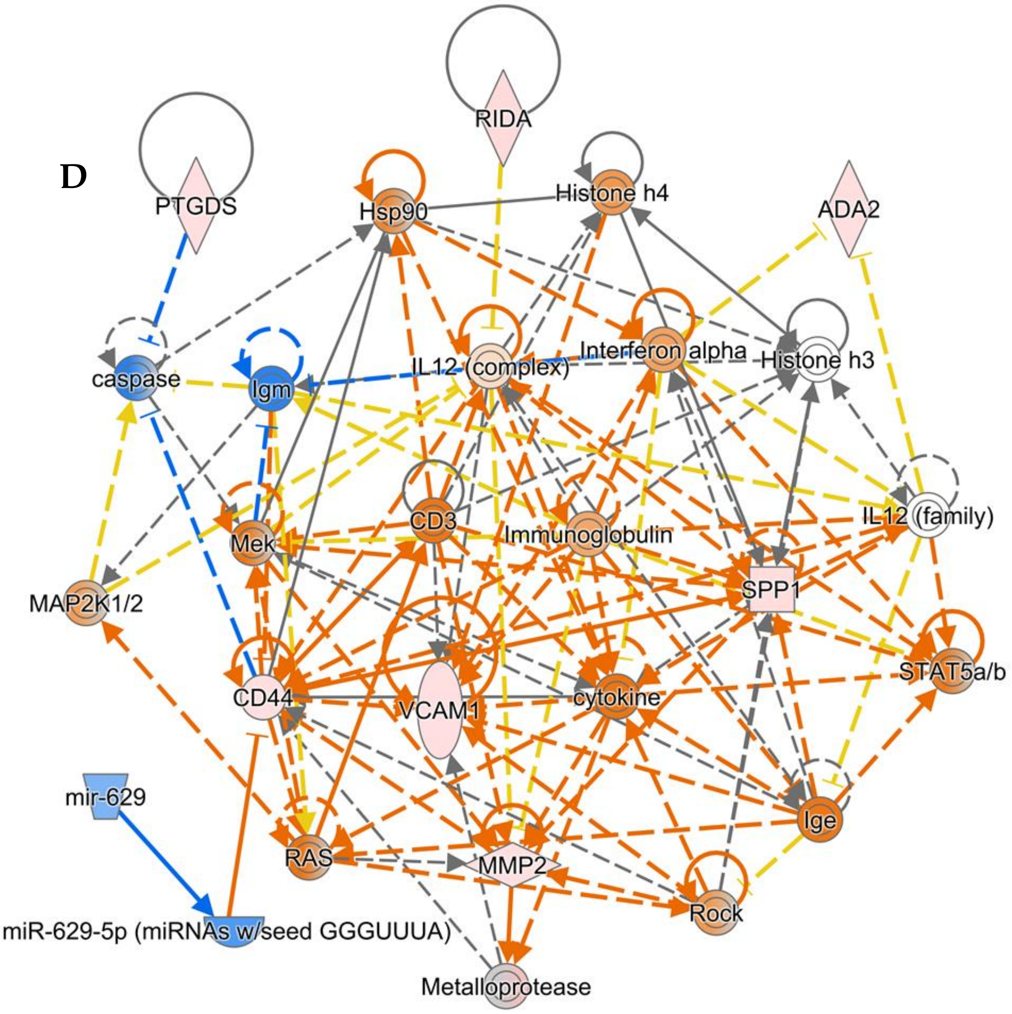

PTGDS
RIDA
Hsp90
Histone h4
ADA2
caspase
Igm
IL12 (complex)
Interferon alpha
Histone h3
CD3
Immunoglobulin
IL12 (family)
Mek
MAP2K1/2
SPP1
STAT5a/b
CD44
VCAM1
cytokine
mir-629
Ige
RAS
MMP2
Rock
miR-629-5p (miRNAs w/seed GGGUUUA)
Metalloprotease

E

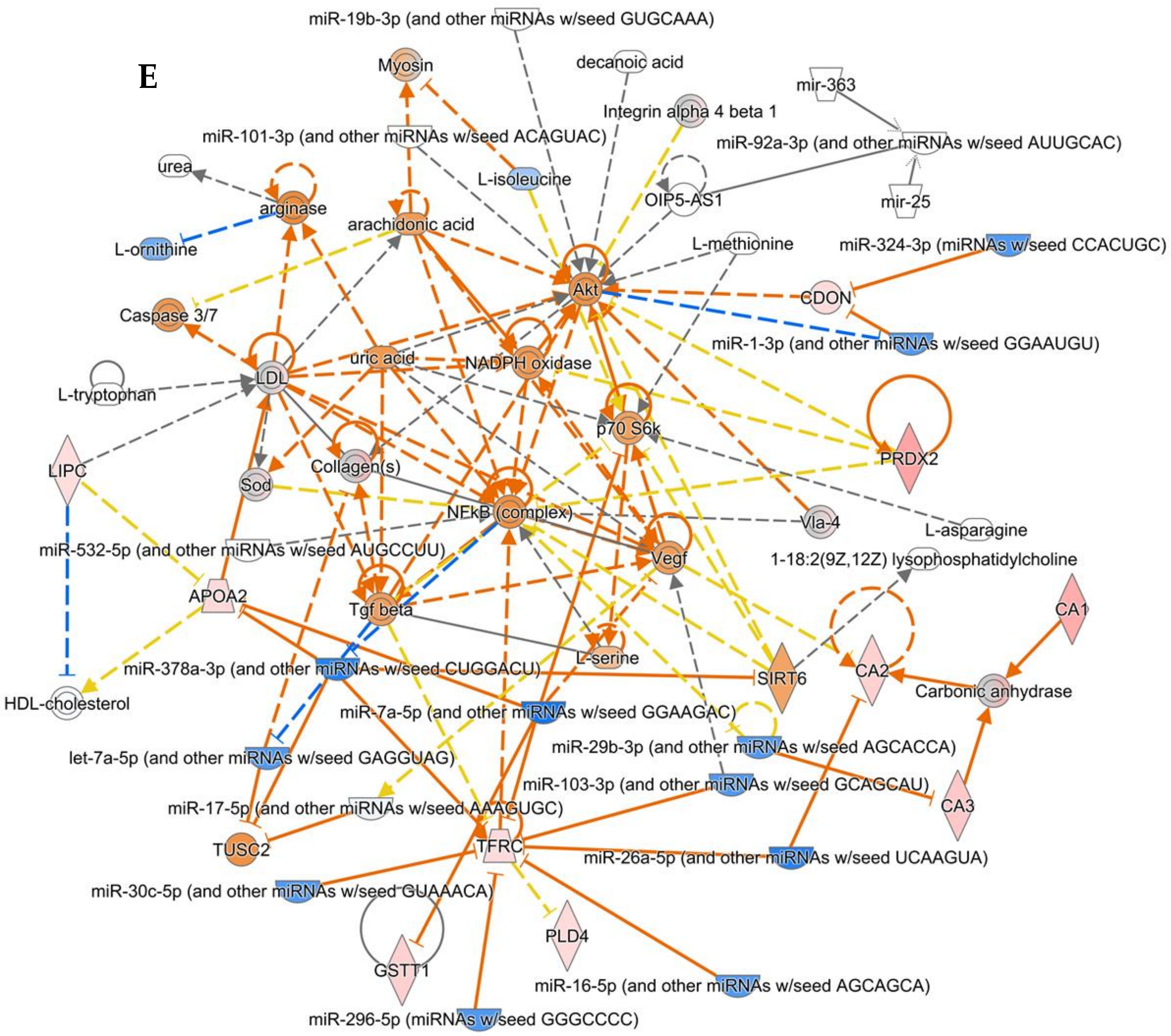
miR-19b-3p (and other miRNAs w/seed GUGCAAA)
Myosin
decanoic acid
mir-363
Integrin alpha 4 beta 1
miR-101-3p (and other miRNAs w/seed ACAGUAC)
miR-92a-3p (and other miRNAs w/seed AUUGCAC)
urea
L-isoleucine
arginase
OIP5-AS1
mir-25
arachidonic acid
L-ornithine
L-methionine
miR-324-3p (miRNAs w/seed CCACUGC)
Akt
CDON
Caspase 3/7
miR-1-3p (and other miRNAs w/seed GGAAUGU)
uric acid
NADPH oxidase
L-tryptophan
LDL
p70 S6k
PRDX2
LIPC
Collagen(s)
Sod
NFkB (complex)
Vla-4
L-asparagine
miR-532-5p (and other miRNAs w/seed AUGCCUU)
Vegf
1-18:2(9Z,12Z) lysophosphatidylcholine
APOA2
CA1
Tgf beta
L-serine
CA2
miR-378a-3p (and other miRNAs w/seed CUGGACU)
SIRT6
Carbonic anhydrase
HDL-cholesterol
miR-7a-5p (and other miRNAs w/seed GGAAGAC)
miR-29b-3p (and other miRNAs w/seed AGCACCA)
let-7a-5p (and other miRNAs w/seed GAGGUAG)
miR-103-3p (and other miRNAs w/seed GCAGCAU)
CA3
miR-17-5p (and other miRNAs w/seed AAAGUGC)
TUSC2
TFRC
miR-26a-5p (and other miRNAs w/seed UCAAGUA)
miR-30c-5p (and other miRNAs w/seed GUAAACA)
PLD4
GSTT1
miR-16-5p (and other miRNAs w/seed AGCAGCA)
miR-296-5p (miRNAs w/seed GGGCCCC)

F

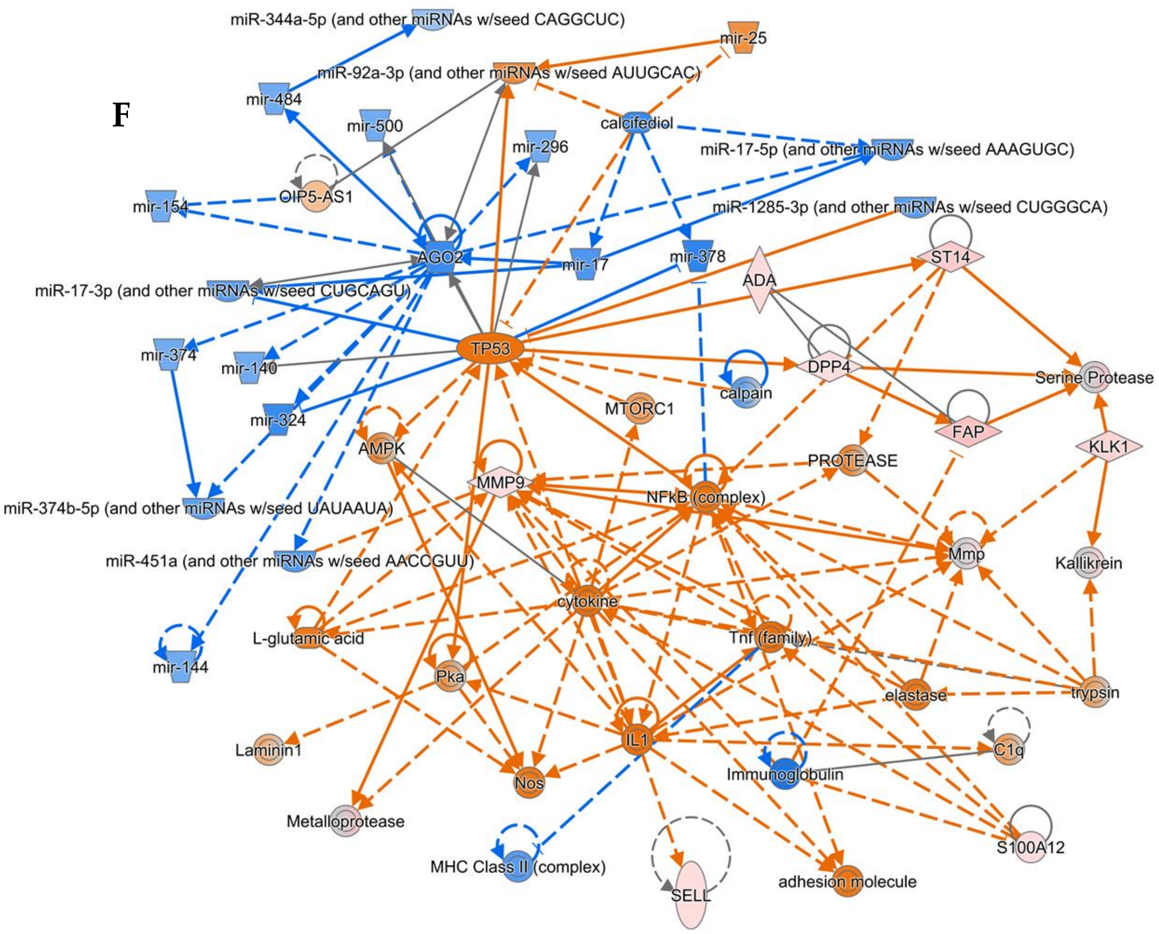
miR-344a-5p (and other miRNAs w/seed CAGGCUC)
mir-25
miR-92a-3p (and other miRNAs w/seed AUUGCAC)
mir-484
mir-500
calcifediol
miR-17-5p (and other miRNAs w/seed AAAGUGC)
mir-296
mir-154
OIP5-AS1
miR-1285-3p (and other miRNAs w/seed CUGGGCA)
AGO2
mir-17
mir-378
ST14
ADA
miR-17-3p (and other miRNAs w/seed CUGCAGU)
TP53
mir-374
mir-140
DPP4
calpain
Serine Protease
mir-324
MTORC1
FAP
AMPK
PROTEASE
KLK1
MMP9
NFkB (complex)
miR-374b-5p (and other miRNAs w/seed UAUAAUA)
miR-451a (and other miRNAs w/seed AACCGUU)
Mmp
Kallikrein
cytokine
L-glutamic acid
Tnf (family)
mir-144
Pka
elastase
trypsin
IL1
Laminin1
C1q
Immunoglobulin
Nos
Metalloprotease
S100A12
MHC Class II (complex)
SELL
adhesion molecule

G

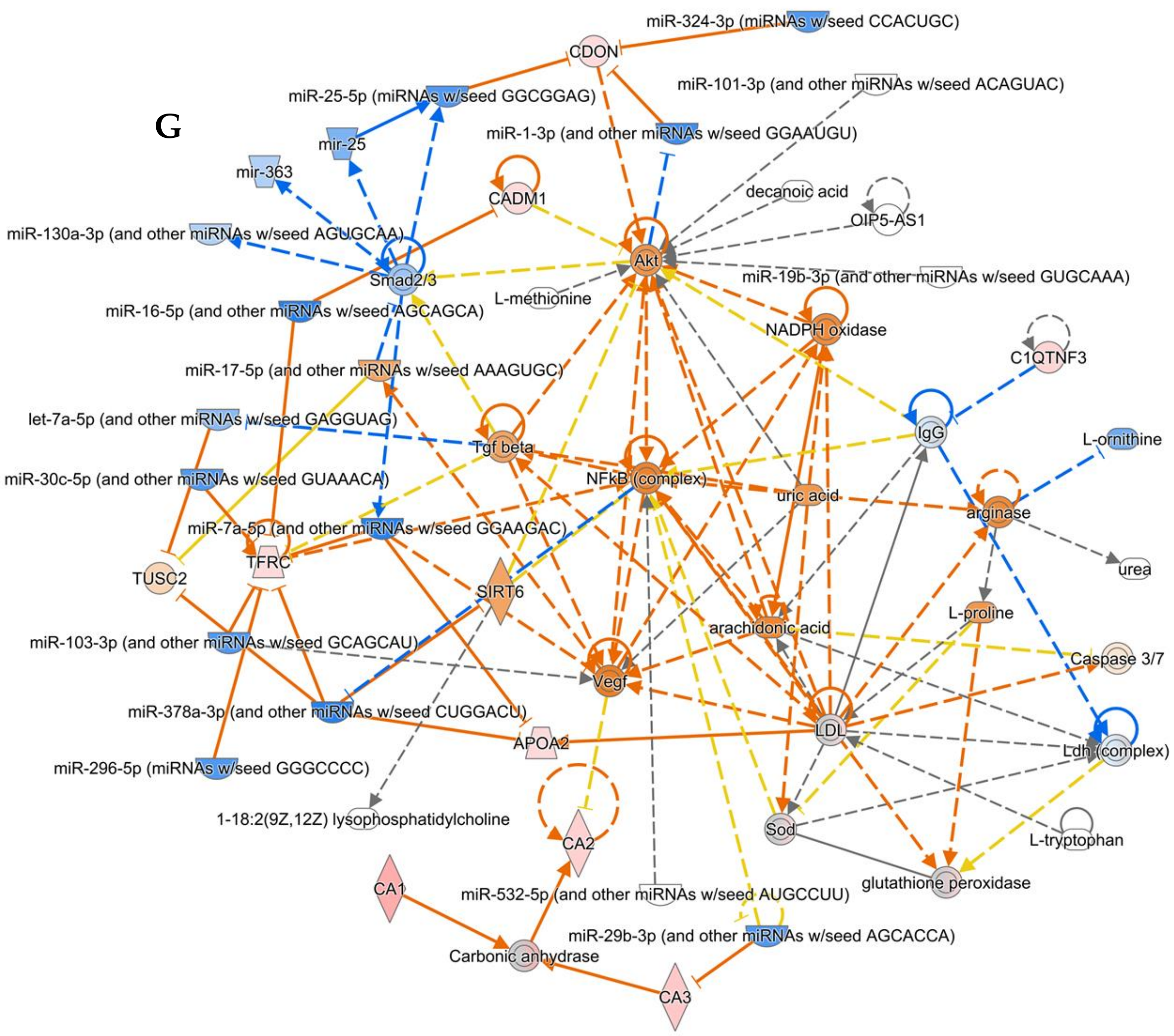
miR-324-3p (miRNAs w/seed CCACUGC)
CDON
miR-101-3p (and other miRNAs w/seed ACAGUAC)
miR-25-5p (miRNAs w/seed GGCGGAG)
mir-25
miR-1-3p (and other miRNAs w/seed GGAAUGU)
mir-363
CADM1
decanoic acid
OIP5-AS1
miR-130a-3p (and other miRNAs w/seed AGUGCAA)
Akt
Smad2/3
L-methionine
miR-19b-3p (and other miRNAs w/seed GUGCAAA)
miR-16-5p (and other miRNAs w/seed AGCAGCA)
NADPH oxidase
C1QTNF3
miR-17-5p (and other miRNAs w/seed AAAGUGC)
let-7a-5p (and other miRNAs w/seed GAGGUAG)
IgG
Tgf beta
L-ornithine
NFkB (complex)
miR-30c-5p (and other miRNAs w/seed GUAAACA)
uric acid
arginase
miR-7a-5p (and other miRNAs w/seed GGAAGAC)
TUSC2
TFRC
urea
SIRT6
L-proline
arachidonic acid
miR-103-3p (and other miRNAs w/seed GCAGCAU)
Caspase 3/7
Vegf
miR-378a-3p (and other miRNAs w/seed CUGGACU)
LDL
APOA2
Ldh (complex)
miR-296-5p (miRNAs w/seed GGGCCCC)
1-18:2(9Z,12Z) lysophosphatidylcholine
Sod
CA2
L-tryptophan
CA1
glutathione peroxidase
miR-532-5p (and other miRNAs w/seed AUGCCUU)
miR-29b-3p (and other miRNAs w/seed AGCACCA)
Carbonic anhydrase
CA3

H

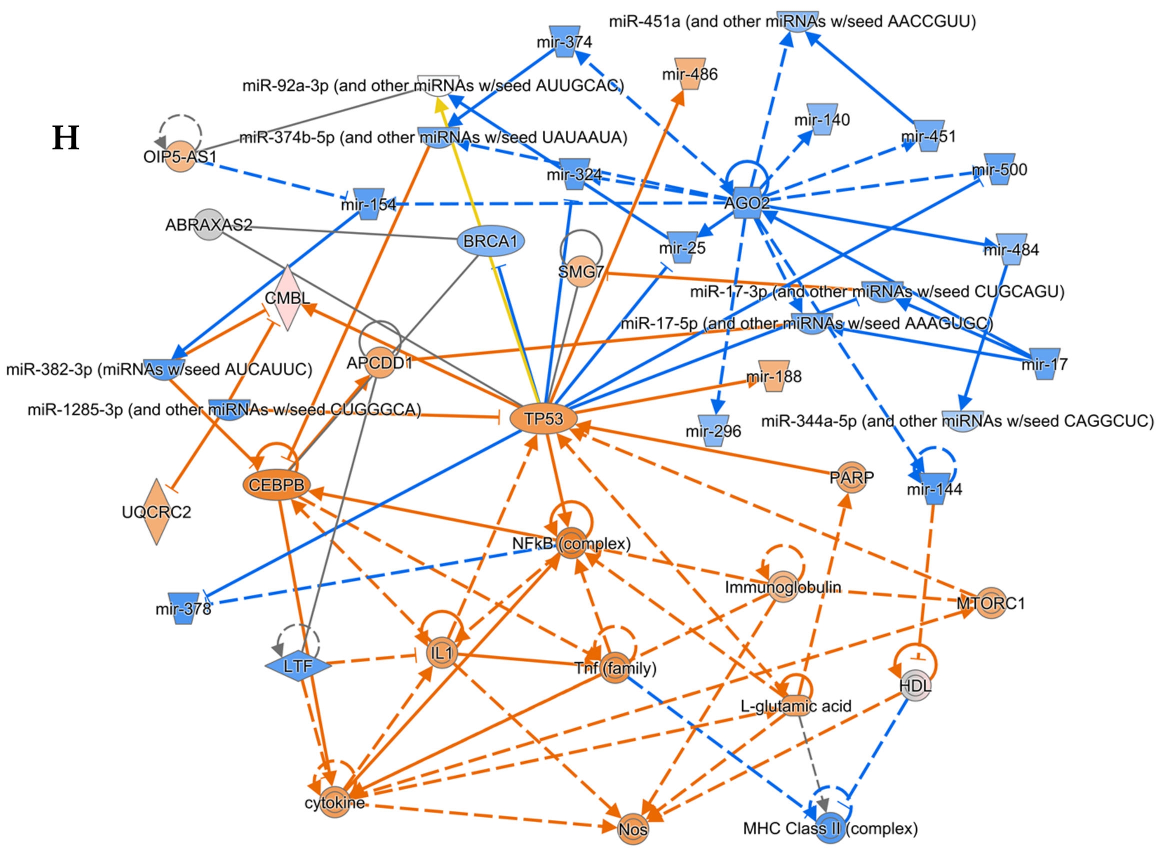
miR-451a (and other miRNAs w/seed AACCGUU)
mir-374
mir-486
miR-92a-3p (and other miRNAs w/seed AUUGCAC)
mir-140
mir-451
OIP5-AS1
miR-374b-5p (and other miRNAs w/seed UAUAAUA)
mir-324
mir-500
mir-154
AGO2
ABRAXAS2
BRCA1
mir-25
mir-484
SMG7
CMBL
miR-17-3p (and other miRNAs w/seed CUGCAGU)
miR-17-5p (and other miRNAs w/seed AAAGUGC)
APCDD1
miR-382-3p (miRNAs w/seed AUCAUUC)
mir-17
mir-188
miR-1285-3p (and other miRNAs w/seed CUGGGCA)
TP53
mir-296
miR-344a-5p (and other miRNAs w/seed CAGGCUC)
CEBPB
PARP
mir-144
UQCRC2
NFkB (complex)
Immunoglobulin
MTORC1
mir-378
LTF
IL1
Tnf (family)
HDL
L-glutamic acid
cytokine
Nos
MHC Class II (complex)

I

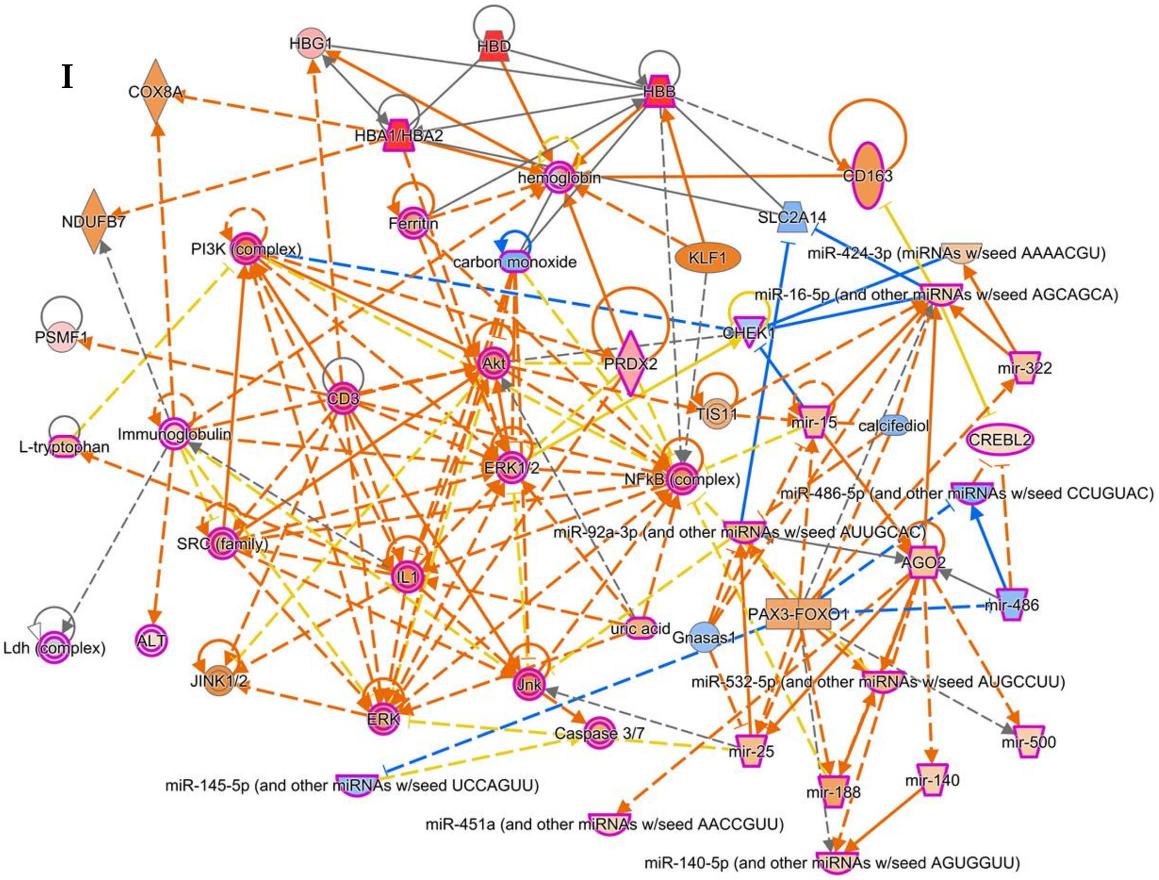
HBG1
HBD
COX8A
HBB
HBA1/HBA2
CD163
hemoglobin
NDUFB7
Ferritin
SLC2A14
PI3K (complex)
KLF1
carbon monoxide
miR-424-3p (miRNAs w/seed AAAACGU)
miR-16-5p (and other miRNAs w/seed AGCAGCA)
PSMF1
CHEK1
Akt
PRDX2
mir-322
CD3
TIS11
mir-15
calcifediol
CREBL2
L-tryptophan
Immunoglobulin
ERK1/2
NFkB (complex)
miR-486-5p (and other miRNAs w/seed CCUGUAC)
miR-92a-3p (and other miRNAs w/seed AUUGCAC)
SRC (family)
AGO2
IL1
PAX3-FOXO1
mir-486
uric acid
Gnasas1
Ldh (complex)
ALT
JINK1/2
Jnk
miR-532-5p (and other miRNAs w/seed AUGCCUU)
ERK
Caspase 3/7
mir-500
mir-25
mir-188
mir-140
miR-145-5p (and other miRNAs w/seed UCCAGUU)
miR-451a (and other miRNAs w/seed AACCGUU)
miR-140-5p (and other miRNAs w/seed AGUGGUU)

J

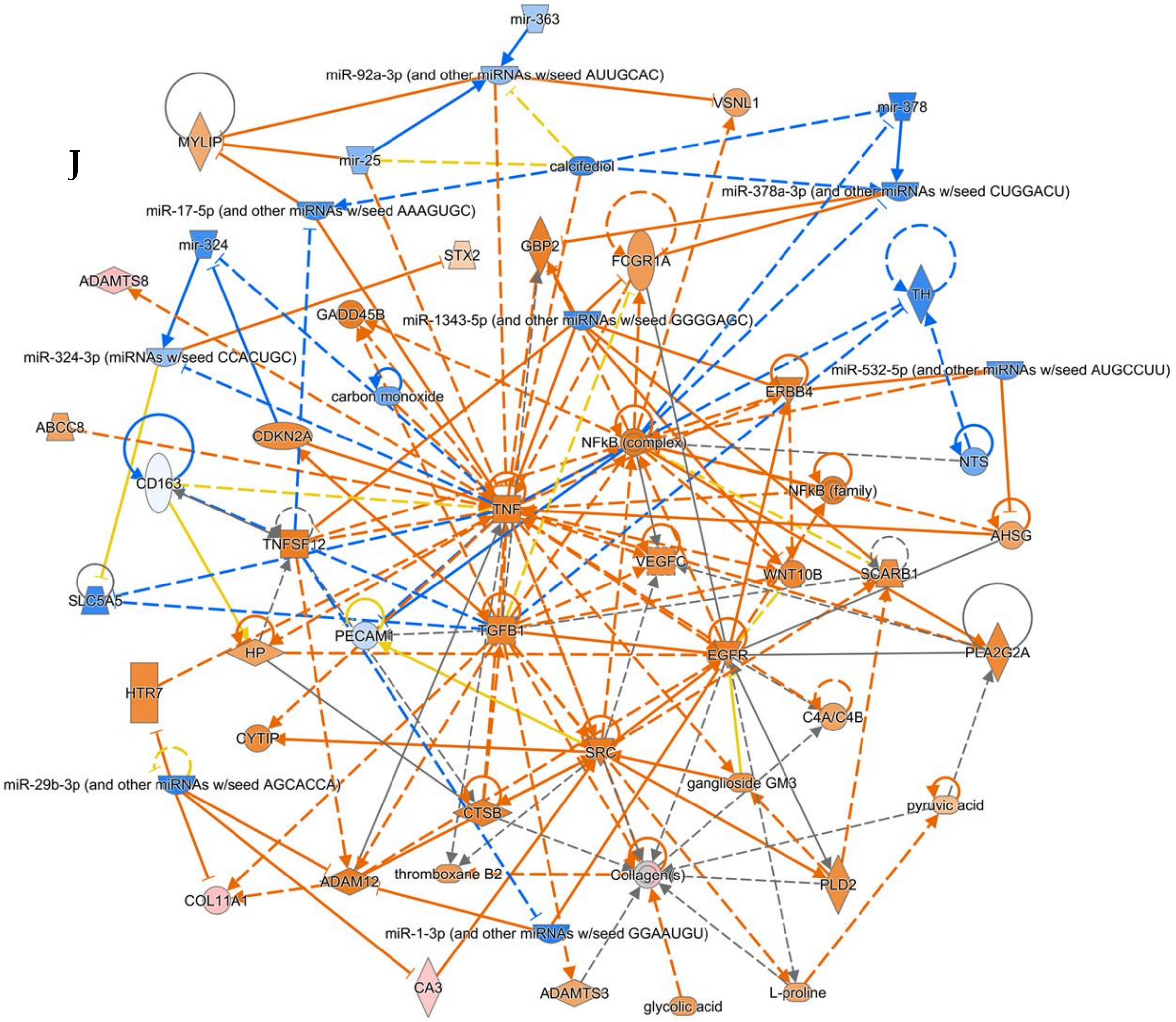

Figure S3 (corresponds to Figure 6). Networks predicted at various stringency levels of feature selection in the analyses shown in Figure 6. Networks predicted at the cutoff values of **(A–D)** 1.1, **(E,F)** 1.2, **(G,H)** 1.3, and **(I,J)** 2.0. See Figure 6 for a summary of key molecules consistently predicted at the different cutoff values in these analyses. Marker shape key – horizontal-oval: transcription regulator; vertical-oval: transmembrane receptor; diamond: enzyme; square: cytokine; vertical-rectangle: G-protein coupled receptor; broken-lined vertical-rectangle: ion channel; horizontal-diamond: peptidase; trapezoid: transporter; concentric circle: complex; circle: other; horizontal ellipse: metabolite; cut ellipsoid: microRNA; and inverted trapezoid: microRNA family. Marker color key – orange: predicted activation, blue: predicted inhibition. Connecting line color key – orange: activation; blue: inhibition; yellow: findings are inconsistent with the state of the downstream molecule; and gray: not predicted.

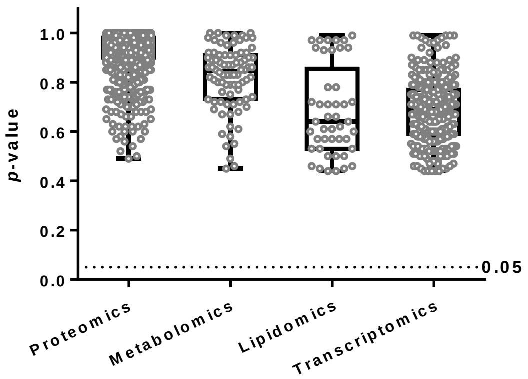

Figure S4. Variance within the high-risk T1D group with and without including the one new-onset subject. Shown are *p*-values of the comparison (by *t*-test) within the high-risk T1D group showing that no significant change ($p < 0.05$; dotted horizontal line) within the group is caused by inclusion of the one new-onset subject. Data points correspond to each analyte/feature identified by each omics approach in all samples without exception.

Table S1. Demographic, serological, and other information of subjects from whom blood samples were obtained in this study. Subjects are divided in two groups, namely the high-risk T1D and healthy (control) subjects, in accordance with the TrialNet staging system for T1D risk.

Subject #	Age (years)	Sex	Race/ Ethnicity	1st degree relatives with T1D	AAbs #	Autoantibodies (years since positive until sample collection)					HLA typing		OGTT
						GAD65/ GAD65H	ICA512/ IA-2H	mIAA	ICA	Znt8	DQA1*0120/DQB1*0602 protective allele	DQB1*0302	
**High-risk T1D subjects**													
1	13	M	White Caucasian	Yes	5	Positive (<1 year)[1]	Positive (<1 year)[1]	Positive (<1 year)[1]	Positive (<1 year)[1]	Positive (<1 year)[1]	ND	ND	Abnormal[2]
2	14	M	White Caucasian	Yes	4[3]	Positive (9 years)	Positive (6 years)	Positive (9 years)	Positive (5 years)	Questionable[3]	Negative	Positive	Normal
3	14	M	White Hispanic	Yes	5	Positive (9 years)	Positive (9 years)	Positive (12 years)	Positive (12 years)	Positive (3 years)	Negative	Negative	Normal
4	11	F	White Caucasian	Yes	5	Positive (6 years)	Positive (3 years)	Positive (5 years)	Positive (6 years)	Positive (6 years)	Negative	Positive	Normal
**Healthy subjects**													
1	22	F	White Caucasian	No	0	Negative	Negative	Negative	Negative	Negative	ND	ND	Normal
2	31	F	White Caucasian	No	0	Negative	Negative	Negative	Negative	Negative	ND	ND	Normal
3	19	M	White Hispanic	No	0	Negative	Negative	Negative	Negative	Negative	ND	ND	Normal
4	34	M	Asian	No	0	Negative	Negative	Negative	Negative	Negative	ND	ND	Normal

[1] Subject confirmed positive for all 5 autoantibodies (AAbs) twice before sample collection for this study. 1st and 2nd positive confirmations were respectively 4 and 1 months prior to sample collection.

[2] Two samples collected for this study two weeks apart, 1st sample collected before OGTT (fasting) and 2nd after OGTT (post-prandial) when diagnosis was confirmed.

[3] ZnT8 was positive in 2017 and then negative in 2018.

Table S2. Transcriptomics data quality control. All samples showed a minimum of 14.65 million passed-filter 75 nt single-end reads in the RNA sequencing. After removal of reads comprised substantially of adaptamer sequences and trimming partial adapter sequences, the reads aligned at an average efficiency of 63% +/- 15% (SD) to the hg38 human reference genome and hg38 RNA database.

High-risk T1D subjects

Subject #	PF Clusters	Yield (MB)	% Perfect Index Reads	% of ≥ Q30 Bases (PF)	Mean Quality Score (PF)	Reads Passing All Filters	Reads Mapped to genome with Mismatch	Reads Mapped / Passed All Filters Read (%)
1*	16202916	1227	93.96%	91.39%	33.823	13878248	6415772	46.23%
1*	14646469	1109	92.76%	84.67%	32.474	12038011	8359355	69.44%
2	27600411	2093	93.24%	89.14%	33.384	23046222	16995316	73.74%
3	27696989	2100	94.04%	90.91%	33.730	22411557	17273116	77.07%
4	20079312	1521	90.63%	78.94%	31.271	17627203	15119578	85.77%

Healthy subjects

Subject #	PF Clusters	Yield (MB)	% Perfect Index Reads	% of ≥ Q30 Bases (PF)	Mean Quality Score (PF)	Reads Passing All Filters	Reads Mapped to genome with Mismatch	Reads Mapped / Passed All Filters Read (%)
1	18547614	1406	95.11%	90.25%	33.595	15982462	7903801	49.45%
2	32940064	2500	91.34%	88.95%	33.313	21716390	14897669	68.60%
3	17248712	1306	92.78%	90.37%	33.610	12379105	6222993	50.27%
4	23922839	1814	85.17%	86.55%	32.808	17548491	8120520	46.27%

* Two samples collected for this study from this subject two weeks apart, 1st sample collected before OGTT (fasting) and 2nd after OGTT (post-prandial) when diagnosis was confirmed

Table S3. Complete list of candidate biomarkers in high-risk T1D predicted by *IPA* in the individual quadra-omics datasets. Biomarker prediction by *IPA* was performed in the individual omics datasets with feature selection threshold at cutoff value of 1.1. The top 10 features (separated by a double line) were selected based on their involvement in central nodes within the global molecular network generated from the combined quadra-omics dataset (also shown in Table 2).

			Expression			
**Feature**	**Accession no.**[1]	**Omics**	**Fold-change**[2]	***p*-value**[3]	***IPA*-predicted Function/Disorder**	**Link to T1D**
let-7a-5p	MIMAT0000062	Transcriptomics	1.834	0.133	Inflammation of organ	
miR-130a-3p	MIMAT0000425	Transcriptomics	1.503	0.745	Chronic inflammatory disorder	
miR-16-5p	MIMAT0000069	Transcriptomics	2.954	0.151	Maturation of monocytes, chronic inflammatory disorder, inflammation of organ	[109]
miR-296-5p	MIMAT0000690	Transcriptomics	1.444	0.696	Inflammation of organ	[110]
miR-30c-5p	MIMAT0000244	Transcriptomics	1.39	0.326	Chronic inflammatory disorder, inflammation of organ	
miR-324-3p	MIMAT0000762	Transcriptomics	2.682	0.468	Inflammation of organ	[108]
miR-532-5p	MIMAT0002888	Transcriptomics	2.484	0.122	Chronic inflammatory disorder, inflammation of organ	
miR-92a-3p	MIMAT0000092	Transcriptomics	21.029	0.146	Induction of follicular T helper precursor cells, chronic inflammatory disorder, inflammation of organ	
Uric acid	69-93-2	Metabolomics	2.008	0.872	Activation of macrophages, immune response of cells, induction of peripheral blood lymphocytes, inflammation of organ	[111]
LPC 1-18:1(11Z)		Lipidomics	1.549	0.00949	None	
HBA1/HBA2	P69905	Proteomics	54.293	0.383	Chronic inflammatory disorder, diabetes mellitus, inflammation of organ, systemic autoimmune syndrome	
miR-3131	MIMAT0014996	Transcriptomics	44.427	0.125	Inflammation of organ	
miR-150-3p	MIMAT0004610	Transcriptomics	5.17	0.181	Chronic inflammatory disorder, inflammation of organ	[115]
miR-486-5p	MIMAT0002177	Transcriptomics	3.268	0.00272	Inflammation of organ	[116]
miR-629-5p	MIMAT0004810	Transcriptomics	2.625	0.0861	Inflammation of organ	
miR-500a-3p	MIMAT0002871	Transcriptomics	2.144	0.561	Inflammation of organ	
Citric acid	5949-29-1	Metabolomics	1.997	0.959	Accumulation of pyruvic acid, inflammation of organ	
D-glucose	50-99-7	Metabolomics	1.997	0.636	Accumulation of pyruvic acid, activation of macrophages, cell viability of islet cells, immune response of cells, proliferation of CD4+ T-lymphocytes, release of arachidonic acid, inflammation of organ	
Urea	57-13-6	Metabolomics	1.986	0.444	Inflammation of organ	
Cholesterol	57-88-5	Metabolomics	1.964	0.153	Activation of macrophages, cell spreading of transitional B lymphocytes, chemotaxis of pro-B lymphocytes, immune response of cells, inflammation of organ, proliferation of CD4+ T-lymphocytes, release of arachidonic acid, ruffling of transitional B lymphocytes, inflammation of organ, systemic inflammatory response syndrome	[117]

COL1A1	P02452	Proteomics	1.811	0.306	Cell movement of T lymphocytes, transmigration of lymphocytes, transmigration of monocytes, chronic inflammatory disorder, diabetes mellitus, diabetic complication, inflammation of organ, systemic autoimmune syndrome	
miR-1285-3p	MIMAT0005876	Transcriptomics	1.753	0.57	Inflammation of organ	
miR-344a-5p	MIMAT0002174	Transcriptomics	1.748	0.191	Inflammation of organ	
CA2	P54289	Proteomics	1.678	0.39	Chronic inflammatory disorder, diabetes mellitus, diabetic complication, inflammation of organ, systemic autoimmune syndrome	[118]
CAT	P04040	Proteomics	1.669	0.412	Diabetes mellitus, inflammation of organ, systemic autoimmune syndrome	[119]
CDH11	P55287	Proteomics	1.464	0.422	Inflammation of organ, chronic inflammatory disorder, diabetes mellitus, diabetic complication, inflammation of organ, systemic autoimmune syndrome	
ALDH1A1	P00352	Proteomics	1.446	0.47	Diabetes mellitus	[120]
LDLR	P01130	Proteomics	1.443	0.532	Cell movement of T lymphocytes, diabetes mellitus, inflammation of organ, quantity of IL-10 in blood, transmigration of lymphocytes, transmigration of monocytes, diabetic complication	[121]
PC 18:2(9Z,12Z)/ 18:2(9Z,12Z)		Lipidomics	1.387	0.186	None	
TFRC	P02786	Proteomics	1.358	0.584	Inflammation of organ, chronic inflammatory disorder, systemic autoimmune syndrome	[122]
FN1	P02751	Proteomics	1.317	0.553	Cell movement of T lymphocytes, transmigration of lymphocytes, transmigration of monocytes, chronic inflammatory disorder, diabetes mellitus, diabetic complication, inflammation of organ, systemic autoimmune syndrome	[123]
BGLAP	P02818	Proteomics	1.311	0.58	Chronic inflammatory disorder, diabetes mellitus, systemic autoimmune syndrome	[124]
MMP9	P14780	Proteomics	1.307	0.582	Cell movement of T lymphocytes, chronic inflammatory disorder, inflammation of organ, quantity of IL-10 in blood, transmigration of monocytes, diabetes mellitus, systemic autoimmune syndrome	[125]

[1] Accession numbers corresponding to mature miRNAs in the miRbase database, proteins in the human SwissProt database (from Uniprot Knowledgebase), and metabolites in the American Chemical Society (CAS = Chemical Abstract Service). Lipids were annotated in the Thermo Scientific proprietary Lipid Search 4.1 database built into their software without access to accession numbers.

[2] Predicted expression fold-change in the high-risk T1D subjects relative to healthy controls.

[3] Non-adjusted *p*-value as obtained from *IPA* software based on comparisons of high-risk T1D subjects vs. healthy controls in each omics-type dataset independently (*t*-test). Only LPC from the lipidomics and miR-486-5p from transcriptomics datasets had $p < 0.05$.
